# Social disparities and inequalities in healthcare access and expenditures among Iranians exposed to sulfur mustard: a national study using spatio-temporal analysis

**DOI:** 10.1186/s12913-023-10352-7

**Published:** 2023-12-13

**Authors:** Seyed-Morteza Hosseini-Shokouh, Mostafa Ghanei, Batool Mousavi, Hassan Bagheri, Mohammadkarim Bahadori, Mohammad Meskarpour-Amiri, Parisa Mehdizadeh

**Affiliations:** 1 Health Management Research Center, Baqiyatallah University of Medical Sciences, Tehran, Iran; 2 Health Management and Economics Research Center, Health Management Research Institute, Iran University of Medical Sciences, Tehran, Iran; 3 Department of Health Services Management, Faculty of Health, Baqiyatallah University of Medical Sciences, Tehran, Iran; 4 Chemical Injuries Research Center, Baqiyatallah University of Medical Sciences, Tehran, Iran; 5 Prevention Department, Janbazan Medical and Engineering Research Center (JMERC), Tehran, Iran

**Keywords:** Healthcare utilization, Sulfur mustard, Socioeconomic inequity, Spatio-temporal analysis, Iran

## Abstract

**Background:**

Sulfur Mustard (SM) is a chemical warfare agent that has serious short-term and long-term effects on health. Thousands of Iranians were exposed to SM during the eight-year Iran-Iraq conflict and permanently injured while the socioeconomic imbalance in their healthcare utilization (HCU) and health expenditures remains. This study aims to describe the HCU of SM-exposed survivors in Iran from 2018 to 2021; identify high-risk areas; and apply an inequality analysis of utilization regarding the socioeconomic groups to reduce the gap by controlling crucial determinants.

**Methods:**

From Oct 2018 to June 2021, the Veterans and Martyrs Affairs Foundation (VMAF) recorded 58,888 living war survivors with eye, lung, and skin ailments. After cleaning the dataset and removing junk codes, we defined 11 HCU-related variables and predicted the HCU for the upcoming years using Bayesian spatio-temporal models. We explored the association of individual-level HCU and determinants using a Zero-inflated Poisson (ZIP) model and also investigated the provincial hotspots using Local Moran’s I.

**Results:**

With ≥ 90% confidence, we discovered eleven HCU clusters in Iran. We discovered that the expected number of HCU 1) rises with increasing age, severity of complications in survivors' eyes and lungs, wealth index (WI), life expectancy (LE), and hospital beds ratio; and 2) decreases with growing skin complications, years of schooling (YOS), urbanization, number of hospital beds, length of stay (LOS) in bed, and bed occupancy rate (BOR). The concentration index (CInd) of HCU and associated costs in age and wealth groups were all positive, however, the signs of CInd values for HCU and total cost in YOS, urbanization, LOS, and Hospital beds ratio groups were not identical.

**Conclusions:**

We observed a tendency of pro-rich inequity and also higher HCU and expenditures for the elderly population. Finally, health policies should tackle potential socioeconomic inequities to reduce HCU gaps in the SM-exposed population. Also, policymakers should allocate the resources according to the hotspots of HCU.

**Supplementary Information:**

The online version contains supplementary material available at 10.1186/s12913-023-10352-7.

## Background

 Exposure to chemical warfare agents results in serious acute and chronic health consequences. Sulfur Mustard (SM) is a cytotoxic and alkylating compound that has been repeatedly used as a chemical weapon in numerous battles. Hundreds of thousands of people have been exposed to SM since the time the German army unleashed it on a large scale during World War I in 1917 [1]. During the Iraq-Iran conflict in 1980s, Iraq often used SM as a weapon to bombard Iranian border towns and made over 400,000 unmasked residents vulnerable to SM [2–4]. Meanwhile, SM exposure is strongly linked to the emergence of serious complications and injuries in eyes [5], lung [6, 7], and skin [8].

Given that over half of the world's population still does not have complete access to basic health care, it is critical to recognize that these services must be provided to achieve Universal Health Coverage (UHC) [9]. Iranian exposed individuals still seek healthcare services in order to preserve or restore good health, as well as to prevent and treat diseases caused by SM. Healthcare Utilization (HCU) is a fine measure of the number of healthcare services individuals consume [10, 11]. Furthermore, target 3.8 of the Sustainable Development Goals (SDG) is to provide health care to all people regardless of their ability to pay and HCU is a key component of this aim [12].

Significant changes were made to the healthcare system in Iran following the Islamic Revolution of 1979. These changes included incorporating provincial health organizations into medical sciences universities and creating a primary health care network. As a result, from 2000 to 2019, Iran increased its Domestic general government health expenditure (GGHE-D) per capita from 188.23 to 429.78 United States Dollar (USD) [13] while 7% of Iran's GDP goes for healthcare [14]. These changes were made to improve health equity and provide essential healthcare services [15]. Inequality in the usage of healthcare services in Iran has been investigated elsewhere [16, 17]. However, the effectiveness of these programs in promoting the HCU of SM-exposed people and declining inequalities remains unknown, even though these programs expand access to basic healthcare services.

HCU of people involved in military conflicts has been investigated in Asia [18–23], America [24–29], and Europe [30–33]. Aside from a few descriptive studies, to our knowledge, there has been no comprehensive study on tracking social inequalities in different types of HCU among SM-exposed citizens in any country. This study aims to 1) provide a comprehensive description of healthcare utilization of SM-exposed survivors in Iran, and its changes from 2018 to 2021; 2); measure the subnational concentration indices in terms of successful HCU to identify high-risk areas; and 3) apply an association analysis of HCU using the personal characteristics of exposed people along with the environment determinants to estimate the adjusted effects controlled for confounding variables.

## Methods

### Study setting

The Veterans and Martyrs Affairs Foundation (VMAF) reported up to 58,888 alive chemically injured individuals who were exposed to SM from 1980 to 1988. The population was followed from October 2018 until June 2021. Based on the latest divisions, Iran consists of 31 provinces and 32 months that define our study's geographical and temporal framework, respectively.

### Data collection

We defined the variables after cleaning the dataset and addressing the missing and misaligned codes. In the insurance system, every person's met healthcare needs is recorded during this time. We categorized the healthcare services into nine groups named as rehabilitation, laboratory, emergency, imaging, pharmacy, nursing, general practitioner visit, inpatient and other.

### Individual-level variables

The available individual-level factors are age (< 50, 50–59, 60–69 or ≥ 70 years old) and the state of complications in eye, lung and skin (for each: none, mild, moderate or severe).

### Provincial variables

The provincial variables included in this research are age, wealth index (WI), years of schooling (YOS), urbanization, life expectancy (LE), number of hospital beds, length of stay (LOS), bed occupancy rate (BOR) and hospital beds ratio.

The socioeconomic variables in above are calculated based on the merged data of Households Income and Expenditure Survey (HIES) in Iran. Wealth index is calculated through principal component analysis of asset-defining variables of households who participated in HIES [34]. Finally, wealth index was divided into three tertiles (Q1:lowest; Q2:middle; Q3:highest). The same categorization was applied to other provincial variables. We gathered the hospital-based variables through Hospital Statistics and Information System (in Persian: AVAB), a web-based platform designed for collecting important information about hospitals in Iran. We finally merged the mentioned datasets containing socioeconomic and hospital-based variables. We will explain the use of final data in Sect. 2.4.

### Spatial autocorrelation analysis

In this study, we used the Local form of the Moran's I statistics, to identify the hotspots of HUC and cost-related variables across 31 Iranian provinces. If the HCU rate in province $i$ is similar to that of its neighbors, then the value of Local Moran's I will be positive, whereas a negative value indicates an unlikely interpretation and hence a heterologous pattern.

Overall, four categories may be used to classify Local Moran's I value: clusters with high HUC and low HUC (High-High and Low-Low, respectively); clusters with high HUC and lowly utilized neighbors (How-Low); and clusters with low HUC and highly utilized neighbors (Low–High). In Sect. 3.3, we will reveal the significant clusters with 90%, 95%, and 99% confidence. All Iran maps are extracted from https://www.openstreetmap.org to draw the figures and reveal the clusters. More detailed explanations of the method are available elsewhere [35].

### Spatio-temporal modeling

To project the HCU trends, we aggregated their values by month and province. In other words, we transformed the individual-level data to unique combinations reflecting mean values of utilization rate, basic insurance, supplementary insurance and total costs in 31 provinces and 32 months (from Oct 2018 to Jun 2021). In the second step, we fitted a Bayesian spatio-temporal model [35]. We used WI, YOS, urbanization, LE, number of hospital beds, LOS, BOR, and hospital beds ratio as the independent variables of these models by merging the aforementioned dataset wih the one preapred in Sect. 2.2.2.

### Zero-inflated Poisson (ZIP) regression model

In order to investigate the association between socio-demographic factors of individuals and their HCU status, all of the individual-level data sources were pooled and enrolled in this step. The statistical analysis was performed using Zero-inflated Poisson (ZIP) regression model since a significant proportion of population recorded zero HCU during the period [36].

We introduce the model formulation as follows:

1 $${Y}_{i}\sim ZIP({y}_{i}|{\lambda }_{i},{\pi }_{i})$$

2 $$P\left({y}_{i}=j\right)=\left\{\begin{array}{c}{\pi }_{i}+\left(1-{\pi }_{i}\right)\mathrm{exp}\left(-{\lambda }_{i}\right), j=0\\ \left(1-{\pi }_{i}\right)\frac{{{\mu }_{it}}^{{y}_{it}}}{{y}_{it}!}\mathrm{exp}\left(-{\lambda }_{i}\right), j>0\end{array}\right.$$

Where ${y}_{i}$ is the observed HCU for $i$-th individual; ${\lambda }_{it}$ is the Poisson distribution parameter which represents the total number of healthcare services used by $i$-th person from October 2018 to June 2021 and ${\pi }_{i}$ is the probability of zero HCU for $i$-th individual during this period.

Age, eye, lung and skin complication are the individual-level predictors of the multiple ZIP regression model. Provincial variables (WI, YOS, urbanization, LE, number of hospital beds, LOS, BOR, and hospital beds ratio) are also entered into the model as the covariates indicating every participant's residence condition to provide adjusted ORs and RRs based on zero-part and count-part of the model, respectively.

### Concentration index

The concentration index (CInd) [37] was used to assess the degree of inequality in HCU and the expenditures associated with WI, YOS, urbanization, LE, number of hospital beds, LOS, BOR, and hospital beds ratio. Covariance between HCU/Costs and the fractional rank of the person ordered by each of the aforementioned variables may be used to determine CInd, where CInd is equal to twice the area between the concentration curve and the line of equality:

3 $$CInd(y)=\frac{2}{\overline{y} }Cov({y}_{i},{R}_{i})$$

Where ${y}_{i}$ is an integer variable indicating the amount of HUC received by i-th individual, $\overline{y }$ indicates the mean of HCU rate; ${R}_{i}$ stands for the fractional rank of the i-th individual by every aforementioned variable and $Cov$ is the covariance function with sampling weights.

A CInd of 0 indicates no inequality associated with the variables presented. Negative CInd values indicate greater HCU/Costs in individuals with lower WI, YOS, urbanization, LE, number of hospital beds, LOS, BOR, or hospital beds ratio. Positive CInd values suggest that persons with greater WI, YOS, urbanization, LE, number of hospital beds, LOS, BOR, or hospital beds ratio have higher HCU or expenditures.

It should be noted that the individual data was utilized to calculate CInd for age, eye, lung and skin complications while the aggregated provincial data was used to calculate CInd for WI, YOS, urbanization, LE, number of hospital beds, LOS, BOR, and hospital beds ratio.

## Results

### Descriptive characteristics

According to our database, of the total of 58,888 SM-exposed survivors evaluated, 39,946 used at least one healthcare service during the period. In total, 1,804,150 services were identified by the insurance company attributable to SM survivors' use. Likewise, we estimate that on average, 16,183 survivors took benefit of HCU and used 56,380 healthcare services every month, leading to a HCU rate of 0.96 (0.94–0.98) per person per month.

### Age

According to Table 1, the total numbers of SM-exposed people for each age category (in years) were as follows: There were 3,457 people (5.87%) aged < 50; 42,683 (72.49%) aged 50–59; 9,838 (16.71%) aged 60–69; and 2,902 (4.93%) aged ≥ 70 years old. During 32 months, < 50 people had the lowest HCU rate with 29.5 (95% CI: 28.16–30.84) per person and 37.9 (95% CI: 36.31–39.49) per person benefiting from HCU, whereas people aged ≥ 70 years old had the highest HCU rate with 38.36 (95% CI: 36.41–40.3) per person and 54.95 (95% CI: 52.5–57.41) per person benefiting from HCU.

**Table 1:** Descriptive characteristics of the population and their HCU status according to age and complications severity in eye, lung and skin

Variable/Category	No. of exposed people (%)	No. of exposed people benefiting from HCU (%)	No. of services (%)	HCU rate (per one person)		HCU rate (per one person benefiting from HCU)	
				**Mean (SD)**	**95% CI**	**Mean (SD)**	**95% CI**
**Age (years)**							
< 50	3,457 (5.87)	2,688 (6.73)	101,874 (5.65)	29.5 (40.22)	28.16–30.84	37.9 (41.95)	36.31–39.49
50–59	42,683 (72.49)	29,066 (72.76)	1,260,611 (69.87)	29.56 (44.23)	29.14–29.98	43.37 (47.66)	42.82–43.92
60–69	9,838 (16.71)	6,167 (15.44)	330,387 (18.31)	33.62 (49.66)	32.64–34.6	53.57 (53.48)	52.24–54.91
≥ 70	2,902 (4.93)	2,025 (5.07)	111,278 (6.17)	38.36 (53.45)	36.41–40.3	54.95 (56.4)	52.5–57.41
**Eye**							
None	50,654 (86.48)	34,061 (85.68)	1,511,524 (84.14)	29.87 (44.46)	29.48–30.26	44.38 (47.89)	43.87–44.89
Mild	7,595 (12.97)	5,419 (13.63)	268,025 (14.92)	35.31 (50.66)	34.17–36.45	49.46 (53.81)	48.03–50.89
Moderate	155 (0.26)	128 (0.32)	6,962 (0.39)	45.21 (55.95)	36.37–54.04	54.39 (57.17)	44.49–64.29
Severe	169 (0.29)	146 (0.37)	9,849 (0.55)	58.28 (71.22)	47.54–69.02	67.46 (72.48)	55.7–79.22
**Lung**							
None	28,135 (49.01)	18,623 (47.66)	751,758 (42.49)	26.75 (40.75)	26.27–27.22	40.37 (44.23)	39.73–41
Mild	23,516 (40.96)	16,209 (41.48)	785,954 (44.42)	33.45 (47.89)	32.84–34.06	48.49 (50.94)	47.7–49.27
Moderate	5,382 (9.38)	3,949 (10.11)	212,240 (11.99)	39.46 (54.14)	38.01–40.9	53.75 (56.79)	51.97–55.52
Severe	375 (0.65)	294 (0.75)	19,491 (1.1)	51.98 (68.86)	45.01–58.95	66.3 (71.41)	58.13–74.46
**Skin**							
None	52,289 (89.74)	35,203 (88.88)	1,582,177 (88.3)	30.29 (45.23)	29.9–30.68	44.94 (48.76)	44.44–45.45
Mild	5,330 (9.15)	3,915 (9.88)	186,124 (10.39)	34.94 (48.37)	33.64–36.24	47.54 (50.84)	45.95–49.13
Moderate	556 (0.95)	414 (1.05)	20,245 (1.13)	36.41 (50.67)	32.2–40.62	48.9 (53.27)	43.77–54.03
Severe	94 (0.16)	77 (0.19)	3,353 (0.19)	35.67 (38.95)	27.8–43.55	43.55 (38.85)	34.87–52.22
**Monthly**	58,880	16,183	56,380	0.96 (2.26)	0.94–0.98	3.48 (3.14)	3.43–3.53
**Total**	58,880	39,946	1,804,150	30.67 (45.51)	30.3–31.04	45.16 (48.94)	44.68–45.64

### Complications severity

Of the total population, 50,654 (86.48%) were classified as having either no eye complications; 7,595 (12.97%) had mild eye complications, while 324 were classified as having either moderate or severe complications in the eyes (0.55% of them). Similarly, 28,135 (49.01%) and 52,289 (89.74%) were respectively categorized as having either no complications in lung and skin; 23,516 (40.96%) and 5,330 (9.15%) having mild lung and skin complications; 5,757 and 650 experienced upper-moderate complications in lung and skin (10.3% and 1.11%, respectively).

People who showed no eye complications had the lowest HCU rate per ordinary and HCU-benefited person (29.87 and 44.38 respectively) while the highest HCU rates were observed among severely injured people from eyes (58.28 and 67.46 respectively). Similarly, while the greatest HCU rates were found in those with severe lung lesions (51.98 and 66.3 per ordinary and HCU-benefitted individual, respectively), the lowest HCU rates were seen in those with no evidence of lung issues (26.75 and 40.37 respectively). People with no skin issues had the lowest HCU rate per person, as predicted (30.29). Surprisingly, the seriously wounded group had the lowest HCU rate among those who received HCU (43.55). In contrast, those with moderate skin complications recorded the greatest HCU rate per person (36.41) and HCU rate per HCU-benefited person (48.9).

The information regarding healthcare costs according to age and complications severity in eye, lung and skin are available in Table 2.

**Table 2:** Mean healthcare costs (with 95% confidence intervals) according to age and complications severity in eye, lung and skin

Variable/Category	Costs per one health service (USD)			Costs per one person (USD)			Costs per one person benefiting from health services (USD)		
	**Basic insurance**	**Supplementary insurance**	**Total**	**Basic insurance**	**Supplementary insurance**	**Total**	**Basic insurance**	**Supplementary insurance**	**Total**
**Age (years)**									
< 50	7.77 (7.27–8.27)	23.89 (22.69–25.1)	33.14 (31.64–34.64)	229.27 (197.87–260.67)	704.94 (628.54–781.34)	977.8 (879.82–1075.77)	294.52 (254.53–334.51)	905.56 (808.75–1002.38)	1256.08 (1132.22–1379.94)
50–59	8.98 (8.8–9.15)	27.58 (27.07–28.1)	38.28 (37.65–38.9)	265.33 (255.43–275.23)	815.38 (788.71–842.05)	1131.47 (1097.48–1165.46)	389.3 (374.99–403.61)	1196.34 (1157.98–1234.69)	1660.11 (1611.41–1708.8)
60–69	10.62 (9.82–11.42)	32.43 (31.13–33.73)	45.19 (43.58–46.81)	357.1 (325.91–388.29)	1090.33 (1025.89–1154.77)	1519.41 (1438.19–1600.63)	569.04 (520.1–617.98)	1737.42 (1638.2–1836.63)	2421.15 (2297.1–2545.21)
≥ 70	11.11 (10.41–11.81)	36 (33.89–38.1)	49.28 (46.8–51.76)	426.14 (388.55–463.73)	1380.86 (1229.41–1532.3)	1890.19 (1713.53–2066.86)	610.48 (558.65–662.32)	1978.2 (1766.45–2189.95)	2707.87 (2463.21–2952.54)
**Eye**									
None	9.24 (9.02–9.46)	28.58 (28.07–29.09)	39.64 (39.02–40.27)	275.97 (266.06–285.88)	853.71 (828.46–878.96)	1184.07 (1152.17–1215.97)	410.02 (395.52–424.53)	1268.4 (1231.69–1305.12)	1759.23 (1713.05–1805.4)
Mild	10 (9.49–10.52)	29.58 (28.62–30.54)	41.34 (40.08–42.59)	353.16 (326.17–380.16)	1044.43 (971.71–1117.15)	1459.53 (1368.46–1550.6)	494.71 (457.56–531.87)	1463.05 (1363.33–1562.76)	2044.53 (1920.32–2168.74)
Moderate	8.27 (6.71–9.82)	35.7 (29.91–41.5)	45.18 (38.55–51.81)	373.69 (179.98–567.41)	1614.08 (965.39–2262.77)	2042.49 (1238.3–2846.68)	449.6 (218.6–680.6)	1941.94 (1173.4–2710.49)	2457.37 (1505.25–3409.49)
Severe	9.64 (7.72–11.56)	35.71 (29.82–41.6)	47.52 (40.74–54.31)	561.82 (313.56–810.09)	2081.06 (1515.88–2646.25)	2769.62 (2012.59–3526.66)	650.33 (365.48–935.18)	2408.9 (1770.55–3047.25)	3205.94 (2350.63–4061.25)
**Lung**									
None	9.33 (9.09–9.56)	28.74 (27.94–29.54)	40.02 (39.06–40.98)	249.47 (238.69–260.24)	768.79 (735.06–802.52)	1070.46 (1028.53–1112.39)	376.5 (360.54–392.46)	1160.27 (1110.29–1210.25)	1615.55 (1553.72–1677.38)
Mild	9.29 (9.05–9.53)	27.79 (27.2–28.39)	38.78 (38.06–39.5)	310.84 (296.09–325.58)	929.68 (893–966.37)	1297.11 (1250.05–1344.16)	450.6 (429.57–471.62)	1347.69 (1295.79–1399.6)	1880.32 (1814.04–1946.6)
Moderate	9.51 (8.35–10.67)	31.08 (29.79–32.37)	42.27 (40.45–44.09)	375.18 (325.05–425.31)	1226.16 (1132–1320.33)	1667.79 (1549.23–1786.36)	511.04 (443.26–578.83)	1670.18 (1544.75–1795.6)	2271.73 (2114.41–2429.05)
Severe	10.41 (9.28–11.53)	47.52 (42.46–52.59)	60.34 (54.24–66.45)	540.84 (403.47–678.21)	2470.04 (1952.51–2987.58)	3136.45 (2504.8–3768.11)	689.85 (518.46–861.25)	3150.56 (2511.84–3789.29)	4000.58 (3223.23–4777.93)
**Skin**									
None	9.29 (9.12–9.45)	28.68 (28.19–29.17)	39.77 (39.18–40.36)	281.31 (272.31–290.3)	868.74 (843.46–894.01)	1204.59 (1172.93–1236.25)	417.44 (404.33–430.55)	1289.15 (1252.44–1325.86)	1787.54 (1741.78–1833.3)
Mild	9.85 (8.55–11.15)	28.81 (27.52–30.11)	40.5 (38.57–42.43)	344.11 (293.41–394.81)	1006.76 (925.43–1088.09)	1415.04 (1305.35–1524.73)	468.22 (399.64–536.79)	1369.86 (1261.43–1478.3)	1925.39 (1779.4–2071.38)
Moderate	9.32 (8.05–10.6)	34.02 (30.9–37.14)	45.37 (41.46–49.28)	339.44 (258.9–419.99)	1238.74 (988.82–1488.66)	1652.01 (1337.38–1966.64)	455.87 (349.97–561.77)	1663.63 (1337.82–1989.43)	2218.64 (1810.03–2627.25)
Severe	8.58 (6.58–10.58)	41.19 (31.56–50.82)	51.54 (40.94–62.14)	306.03 (185.93–426.13)	1469.16 (763.8–2174.51)	1838.51 (1031.16–2645.86)	373.6 (231.21–515.98)	1793.51 (948.64–2638.39)	2244.41 (1281.26–3207.57)
**Total**	9.34 (9.14–9.54)	28.78 (28.33–29.24)	39.93 (39.38–40.49)	286.47 (277.22–295.73)	882.71 (858.9–906.53)	1224.67 (1194.62–1254.72)	421.88 (408.45–435.31)	1299.94 (1265.62–1334.26)	1803.53 (1760.42–1846.63)

### Healthcare service categories

Among healthcare services, the highest HCU rate was for pharmacy category during the study time (13.1 per person; 20.78 per HCU benefited person). On the contrary, laboratory services had the lowest rate (0.06 per person; 1.39 per HCU benefited person). The greatest and lowest expenses were for inpatient and nursing care (179.21 and 9.94 USD, respectively). The details about other services and their health insurance coverage are presented in Table 3.

**Table 3:** Healthcare utilization and related costs of the population according to healthcare service categories during the period

Service	No. of exposed people benefiting from HCU (%)	No. of services (%)	HCU rate		Costs per one health service (USD)		
			**per one person**	**per one person** **benefiting from HCU**	**Basic insurance**	**Supplementary insurance**	**Total**
Rehabilitation	5,305 (3.05)	10,515 (0.58)	0.18 (0.16–0.19)	1.98 (1.81–2.16)	22.8 (22.28–23.32)	117.76 (115.71–119.82)	148.08 (145.73–150.44)
Laboratory	2,475 (1.42)	3,438 (0.19)	0.06 (0.06–0.06)	1.39 (1.34–1.44)	6.98 (6.6–7.35)	59.72 (57.24–62.2)	68.74 (66.23–71.26)
Emergency	13,179 (7.58)	60,856 (3.37)	1.03 (0.99–1.08)	4.62 (4.45–4.79)	0.91 (0.69–1.13)	13.17 (9.74–16.59)	14.5 (11.07–17.94)
Imaging	26,043 (14.98)	89,160 (4.94)	1.52 (1.5–1.54)	3.42 (3.38–3.47)	10.34 (9.78–10.9)	47.68 (45.48–49.88)	59.89 (57.46–62.33)
Pharmacy	37,085 (21.33)	770,788 (42.72)	13.1 (12.94–13.27)	20.78 (20.56–21.01)	12 (11.61–12.39)	26.48 (25.77–27.19)	39.98 (39.06–40.89)
Nursing	17,533 (10.09)	60,156 (3.33)	1.02 (1–1.05)	3.43 (3.36–3.5)	0.51 (0.39–0.62)	8.93 (8.44–9.42)	9.94 (9.41–10.46)
General practitioner visit	36,215 (20.83)	674,459 (37.38)	11.47 (11.32–11.62)	18.62 (18.41–18.83)	2.54 (2.4–2.69)	9.73 (9.4–10.07)	12.64 (12.25–13.04)
Inpatient	29,472 (16.95)	121,660 (6.74)	2.07 (2.04–2.1)	4.13 (4.08–4.18)	37.68 (36.37–38.98)	131.58 (127.8–135.36)	179.21 (174.62–183.8)
Other	6,542 (3.76)	13,118 (0.73)	0.22 (0.21–0.24)	2.01 (1.89–2.12)	2.45 (1.41–3.5)	145.63 (134.93–156.33)	174.23 (162.46–186)

### Subnational estimates

**Fig. 1:**
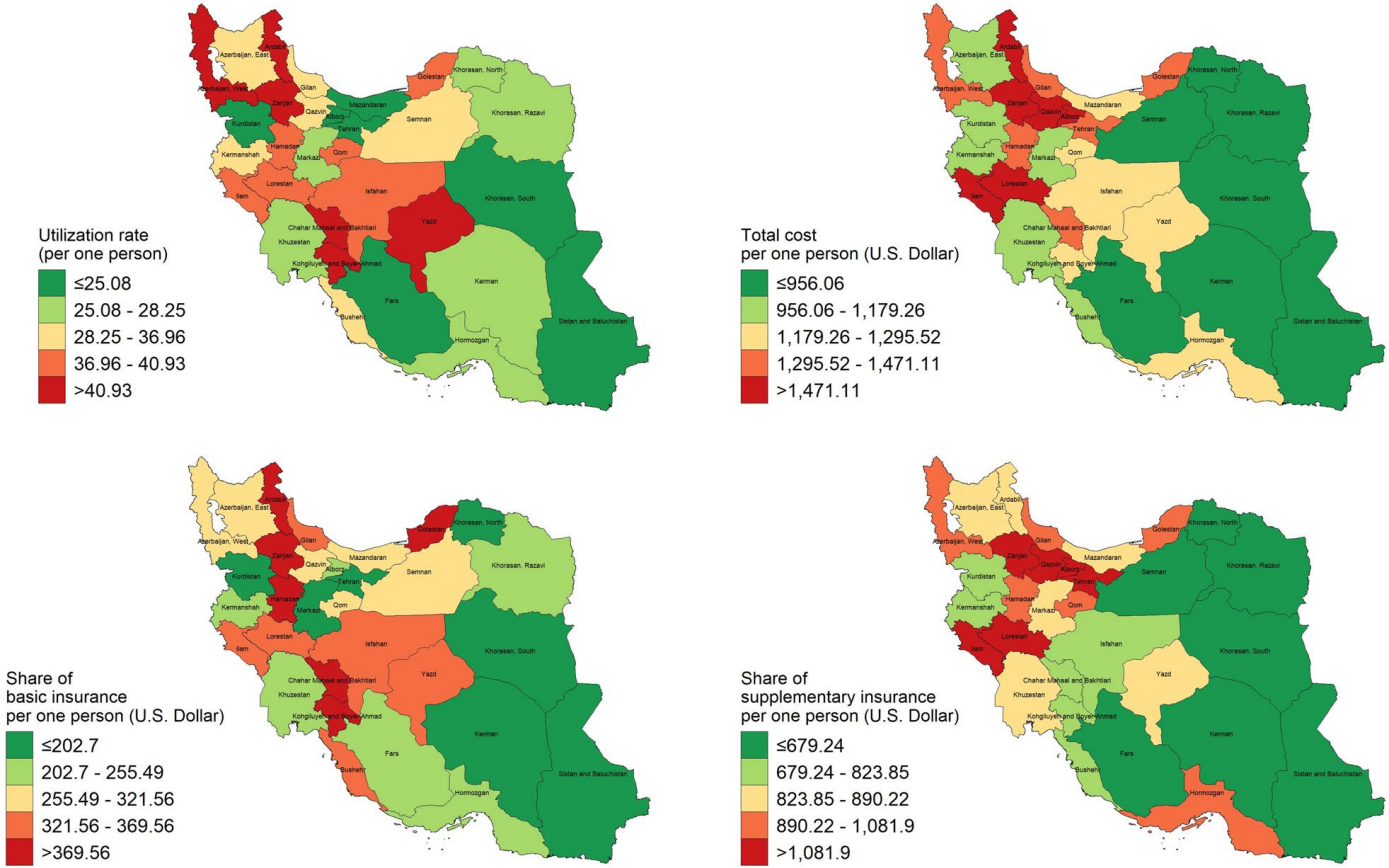
Geographical distribution of HCU and costs paid by insurance plans per one person (Iran’s map is extracted from https://www.openstreetmap.org and further used to depict the figure)

The sparsely-populated and overcrowded residences of SM survivors were respectively in Hormozgan (212) and Tehran (6,751) provinces. Among those who benefited from HCU during this period, *Hormozgan* and *Isfahan* had the lowest (139) and highest (3,891) number of residents, respectively. *Sistan and Baluchistan* and *Chahar Mahaal and Bakhtiari* had the lowest and highest 32-months-HCU rates overall, with rates of 15.2 (95% CI: 12.77–17.63) and 47.17 (95% CI: 43.99–50.34) per person, respectively (Fig. 1). After the data on HCU beneficiaries was filtered, *Kohgiluyeh and Boyer-Ahmad* province had the highest 32-months-HCU rate at 60.96 (95% CI: 58.59–63.34), while *Khorasan, South* had the lowest at 26.61 (95% CI: 23.58–29.65). Other information regarding the HCU status at the subnational level is summarized in Supplementary Table 1.

**Fig. 2:**
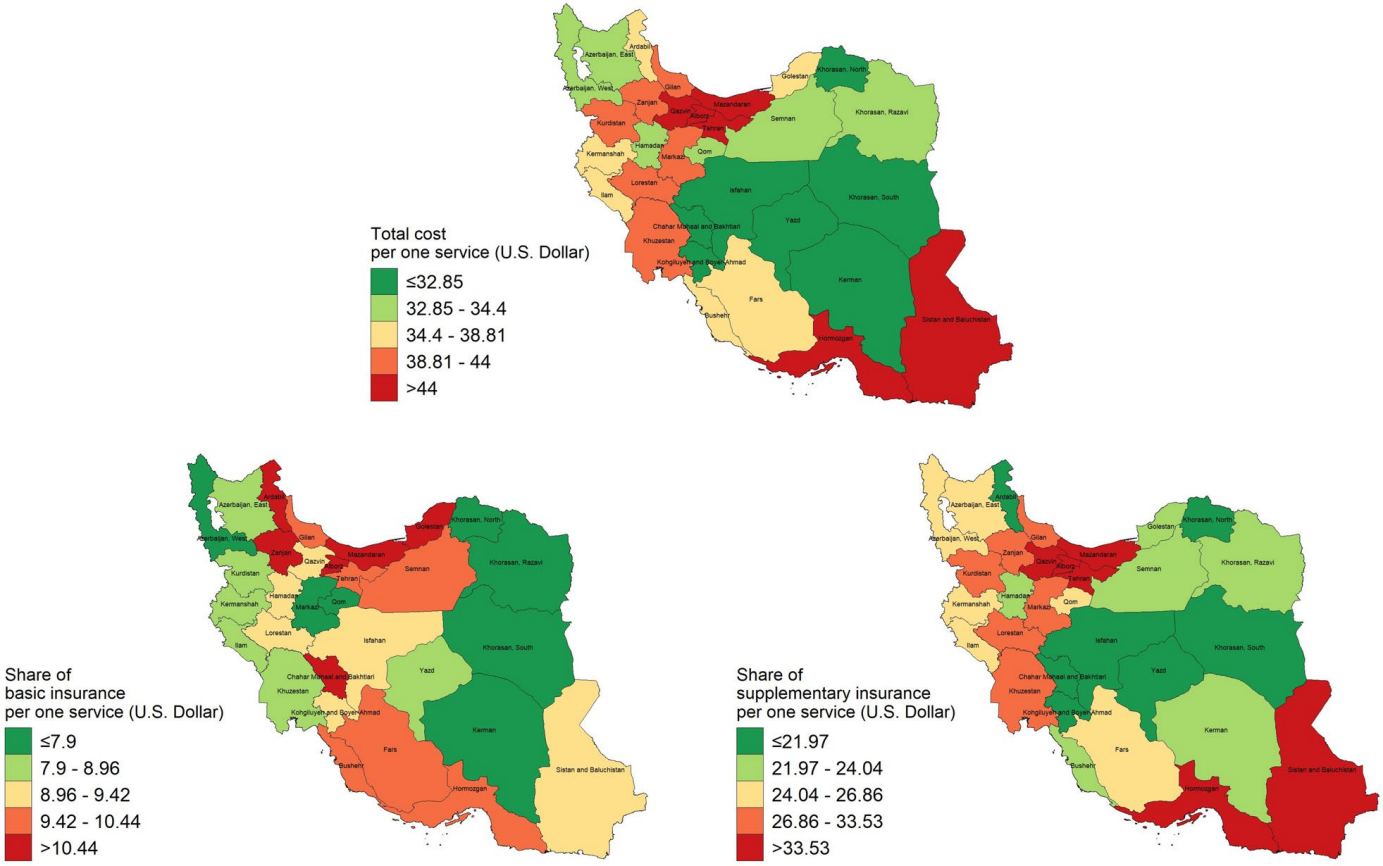
Geographical distribution of HCU and costs paid by insurance plans per one healthcare service (Iran’s map is extracted from https://www.openstreetmap.org and further used to depict the figure)

Figure 2 displays the geographical distribution of paid expenditures by insurance plans per one healthcare service. *Khorasan, North* and *Alborz* had the lowest and highest costs per service, with 26.03 USD (95% CI: 23.31–28.76) and 93.54 USD (95% CI: 84.5–102.57), respectively. During 32 months, we calculated that the total cost-per-person was lowest in *Khorasan, South* at 558.72 USD (95% CI: 440.22–677.22) and highest in *Qazvin* at 1,985.6 USD (95% CI: 1,191.48–2,779.72). *Khorasan, South* also had the lowest cost-per-person after zero-HCU recipients were removed from the data (782.21 USD; 95% CI: 623.64–940.78), while Alborz had the highest (2,935.91 USD; 95% CI: 2,361.34–3,510.48). Supplementary Table 2 provides further data on insurance coverage by subnational area.

### Hotspot analysis

**Fig. 3:**
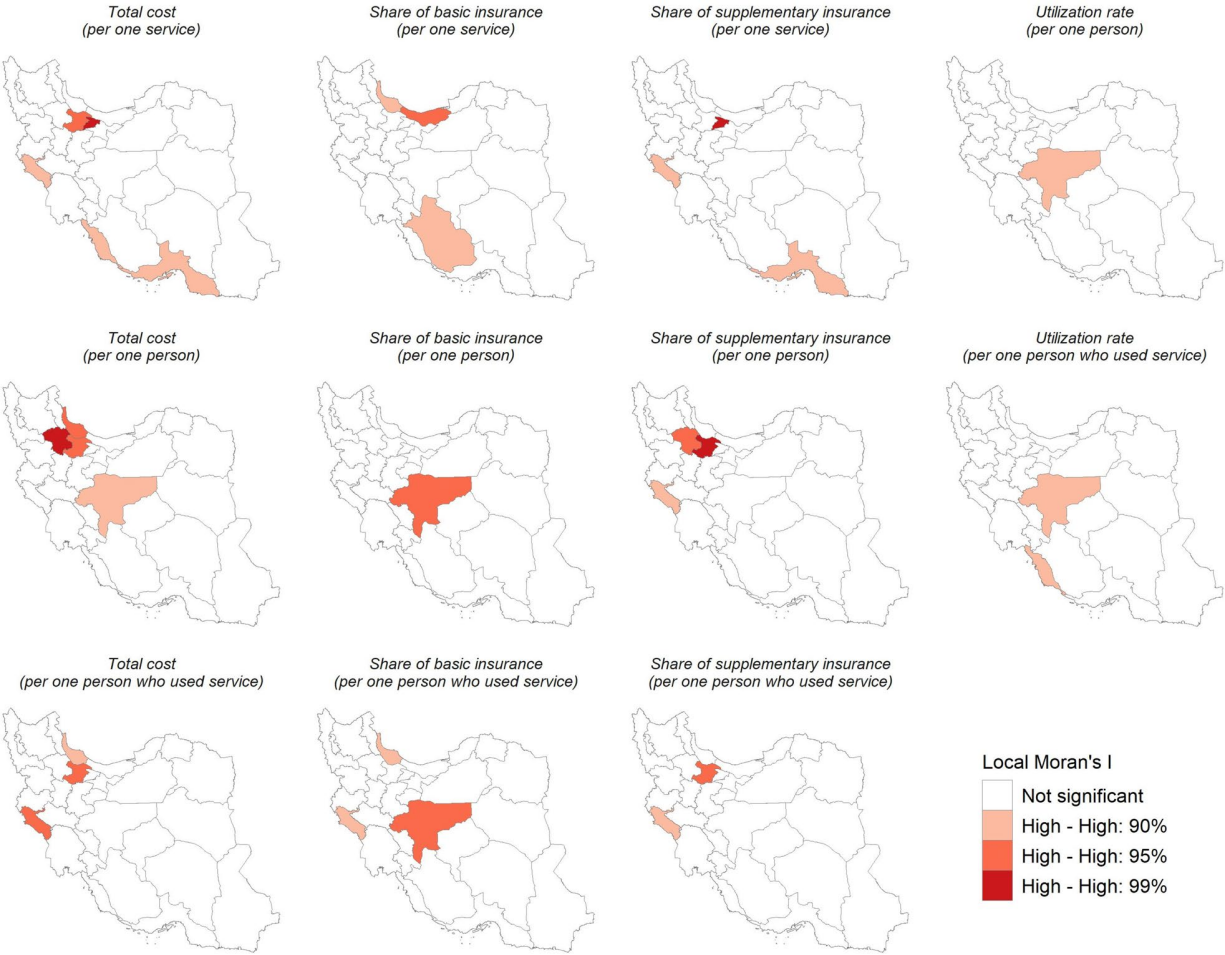
Hotspots of HCU and costs paid by insurance plans in Iran from Nov 2018 to Jun 2021 using Anselin Local Moran's I statistics (Iran’s map is extracted from https://www.openstreetmap.org and further used to depict the figure)

Based on all HCU-related variables, we identified eleven clusters in Iran with at least 90% confidence (Fig. 3). Local Moran's I identified no Low-Low, High-Low, or Low–High clusters, but only High-High (HH). HH clusters are provinces with a high value that have nearby provinces with a high value based on the corresponding index. The most extensive HH clusters were found in Central and Western Iran.

### Association analysis

Table 4 represents the results of ZIP model reflecting the relationship between determinants and HCU of SM-exposed survivors. The findings of the count and inflated zeros of HCU in SM-exposed survivors are shown in the RR and OR columns, respectively. The expected number of HCU rose with increasing age, severity of complications in survivors' eyes and lungs, WI, LE, and Hospital beds ratio (with other factors being constant) (see RRs). Expected HCU, on the other hand, decreases with rising skin complications, YOS, urbanization, number of hospital beds, LOS, and BOR. A person with severe lung injury, for example, is expected to have 1.56 times the number of HCU in a person with no complications (assuming no change in other covariates). In other words, they obtain 56% more HCU than the people with no complications (reference group) under the identical settings.

**Table 4:** Association of determinants with HCU of Iranian people exposed to SM using the ZIP regression model

Variable	Category	RR (p-value)	OR (p-value)	Variable	Category	RR (p-value)	OR (p-value)
Age (years)	< 50	1 (Ref.)	1 (Ref.)	Urbanization rate	Q1 (Lowest)	1 (Ref.)	1 (Ref.)
	50–59	1.138 (< 0.001)	1.488 (< 0.001)		Q2 (Middle)	1.019 (< 0.001)	0.874 (< 0.001)
	60–69	1.398 (< 0.001)	1.949 (< 0.001)		Q3 (Highest)	0.975 (< 0.001)	1.23 (< 0.001)
	≥ 70	1.431 (< 0.001)	1.479 (< 0.001)	Life expectancy (LE)	Q1 (Lowest)	1 (Ref.)	1 (Ref.)
Eye complication	None	1 (Ref.)	1 (Ref.)		Q2 (Middle)	0.906 (< 0.001)	1.227 (< 0.001)
	Mild	1.079 (< 0.001)	0.844 (< 0.001)		Q3 (Highest)	1.135 (< 0.001)	0.884 (0.004)
	Moderate	1.062 (< 0.001)	0.537 (0.006)	Number of hospital beds	Q1 (Lowest)	1 (Ref.)	1 (Ref.)
	Severe	1.375 (< 0.001)	0.363 (< 0.001)		Q2 (Middle)	0.985 (< 0.001)	1.35 (< 0.001)
Lung complication	None	1 (Ref.)	1 (Ref.)		Q3 (Highest)	0.854 (< 0.001)	1.426 (< 0.001)
	Mild	1.181 (< 0.001)	0.899 (< 0.001)	Length of stay (LOS)	Q1 (Lowest)	1 (Ref.)	1 (Ref.)
	Moderate	1.289 (< 0.001)	0.758 (< 0.001)		Q2 (Middle)	1.1 (< 0.001)	0.967 (0.44)
	Severe	1.56 (< 0.001)	0.579 (< 0.001)		Q3 (Highest)	0.8 (< 0.001)	0.998 (0.952)
Skin complication	None	1 (Ref.)	1 (Ref.)	Bed occupancy rate (BOR)	Q1 (Lowest)	1 (Ref.)	1 (Ref.)
	Mild	0.993 (0.007)	0.805 (< 0.001)		Q2 (Middle)	0.787 (< 0.001)	0.842 (< 0.001)
	Moderate	0.98 (0.005)	0.824 (0.059)		Q3 (Highest)	0.877 (< 0.001)	1.155 (< 0.001)
	Severe	0.812 (< 0.001)	0.62 (0.083)	Hospital beds ratio	Q1 (Lowest)	1 (Ref.)	1 (Ref.)
Wealth index (WI)	Q1 (Lowest)	1 (Ref.)	1 (Ref.)		Q2 (Middle)	1.247 (< 0.001)	1.186 (< 0.001)
	Q2 (Middle)	1.021 (< 0.001)	1.043 (0.291)		Q3 (Highest)	1.057 (< 0.001)	0.87 (0.002)
	Q3 (Highest)	1.041 (< 0.001)	0.829 (< 0.001)				
Years of schooling (YOS)	Q1 (Lowest)	1 (Ref.)	1 (Ref.)				
	Q2 (Middle)	0.966 (< 0.001)	1.337 (< 0.001)				
	Q3 (Highest)	0.842 (< 0.001)	1.498 (< 0.001)				

### Inequality analysis

Table 5 presents the concentration index of HCU and healthcare expenditures among individuals by every socioeconomic factor. The concentration index of HCU and related costs in age and wealth groups are all significantly positive, indicating a tendency of pro-rich inequity and also higher usage and costs for the elderly population.

**Table 5:** Concentration index values of HCU and total healthcare costs on groups of every socioeconomic variable

Variable	Concentration index (95% CI)	
	HCU	Total costs
Age	0.02 (0.013,0.027)*	0.081 (0.067,0.096) *
Wealth index (WI)	0.049 (0.042,0.056) *	0.018 (0.005,0.031) *
Years of schooling (YOS)	-0.056 (-0.062,-0.049) *	0.027 (0.013,0.042) *
Urbanization rate	-0.036 (-0.043,-0.029) *	0.028 (0.014,0.043) *
Life expectancy (LE)	-0.012 (-0.02,-0.005) *	-0.004 (-0.018,0.01)
Number of hospital beds	-0.086 (-0.093,-0.08) *	-0.008 (-0.023,0.006)
Length of stay (LOS)	-0.027 (-0.033,-0.02) *	0.017 (0.004,0.03) *
Bed occupancy rate (BOR)	-0.038 (-0.045,-0.031) *	0.012 (-0.002,0.026)
Hospital beds ratio	0.008 (0.001,0.015) *	-0.017 (-0.03,-0.004) *

^*^statistically significant (*p*-value < 0.05)

On the other side, the concentration index values of HCU and costs show different directions based on YOS, urbanization, LOS and Hospital beds ratio. YOS, urbanization, LOS and Hospital beds ratio have negative HCU and positive cost values, implying higher HCU in least educated, urbanized and hospitalized areas while the paid costs are concentrated in most educated, urbanized and hospitalized areas. Inequity according to hospital beds ratio has an opposite pattern, revealing higher HCU in areas that have higher patients proportional to their hospital beds; while the costs are mostly paid by residents of areas where the number of patients is lowest compared to the number of beds.

### Projection

**Fig. 4:**
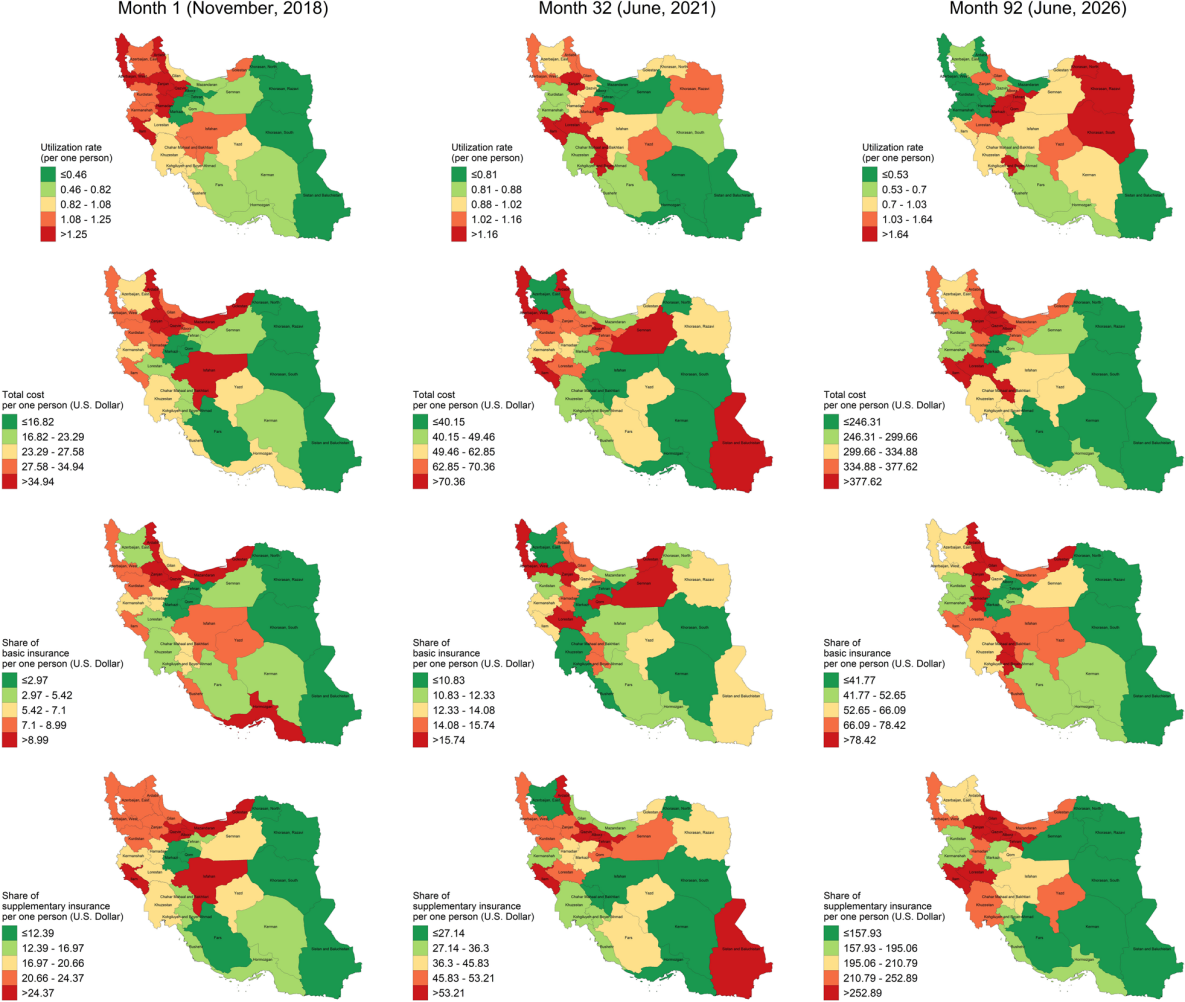
Geographical distribution of HCU and costs paid by insurance plans per one person in different months (Iran’s map is extracted from https://www.openstreetmap.org and further used to depict the figure)

According to the predictions of space–time models, we anticipate that the HCU rate of services will increase by 33.3% from Jun 2021 to Jun 2026 and be equal to 1.24 (0.84–1.69) per person per month. Similarly, the average cost of healthcare services paid per person per month would increase by 4.8-fold and reach 311.23 USD in the next five years. Also, till Jun 2026, the average cost paid by basic and supplementary insurance would be 61.97 USD and 203.81 USD (3.9-fold and 4.2-fold increase), respectively. The spatial distribution regarding the future estimates is displayed in Fig. 4. All the observed and predicted estimates, including the observed and expected payments by insurance plans are available in Supplementary Tables 3, 4, 5.

## Discussion

Although nursing services are the cheapest among healthcare services, only about 10% of HCU received by SM-exposed people are under the nursing category. This might be due to a large amount of informal nursing care. The informal caregivers of SM survivors endure a disproportionally large share of the responsibility for the survivors' care. They often become the primary healthcare providers for their own family members, despite having no formal medical training and little access to financial, emotional, or social support themselves. Caretakers may need to work evenings, weekends, and holidays to meet the 24-h care needs of SM survivors. Aware of the strain imposed on informal caregivers, providers in the health care system should develop solutions to back them up. Nursing professionals should assist in developing interdisciplinary care for disabled SM survivors. The extra guidance might come in the form of supplementary care instructions. Both victims and caregivers should schedule regular check-ups with general practitioners and nurses.

The average cost for receiving one healthcare service was highest in Tehran (the capital of Iran) and surrounding provinces (Fig. 2). It can be justified by the fact that typically high-price services are mostly provided in high-quality medical centers and such centers are more accessible in mega-cities and wealthier divisions. We also assessed this using the concentration index and found a pro-rich inequality in HCU of SM-exposed veterans, which is in line with a number of studies in different countries [38–41].

Unlike LOS and BOR, we found greater HCU in places with more patients relative to their hospital beds, while inhabitants in areas with fewer patients pay the majority of the expenses. The cause might be connected to the Iranian medical insurance policies. Based on UHC, Iran's government launched the Health Sector Evolution Plan (HSEP) to prioritize emergency treatment. Individuals in rural Iran who are only covered by a minimal insurance plan will have the most out-of-pocket expenditures. Besides, high-level hospitals are associated with higher levels of healthcare quality; therefore, patients are more likely to seek treatment in high-level hospitals if they have the option. Consequently, those covered by supplemental insurance firms prefer to seek treatment in high-level hospitals where they believe they will receive the most benefit, resulting in increased medical expenditures and a major pro-rich inequality [42].

The findings also indicate considerable disparities in HCU according to YOS quantiles, implying that low-educated zones have greater HCU. Other researches indicated that less-educated persons use more health resources and have lower health conditions than citizens with higher educational degrees [43, 44]. This might be attributed to the extra healthcare requirements of people with such characteristics that have led to an excess HCU. Higher-educated survivors are better able to manage their own health, adopt healthier habits, and make effective use of healthcare resources because of their increased awareness of these topics and their ability to apply this information. Some diseases, including diabetes and heart disease, may be avoided or kept under control with the support of a healthy lifestyle. Also, mild to moderate SM complications in vital organs may be avoided or mitigated from becoming severe by prompt diagnosis and treatment [45, 46].

The CInd representing HCU equity based on residing area urbanization was negative, indicating that SM-exposed survivors in less urbanized provinces used more care than inhabitants in more urbanized provinces. This gap might be explained by a decrease in low-cost services (e.g., family practitioners) after the implementation of HSEP, which has resulted in excess HCU by rural inhabitants. Although the costs of family doctor appointments in rural Iran have decreased generally, some low-income people still cannot afford them. Most people with basic insurance seek outpatient treatment in village clinics. This might also justify the positive CInd value we estimated for health expenditures based on the urbanization index.

The primary goal of UHC programs is to remove financial obstacles to accessing healthcare for everybody. HSEP was Iran's UHC program established in May 2014 to guarantee citizens access to high-quality medical treatment [47]. In this program, various measures were taken to expand access to basic health insurance, improve the quality of care provided by hospitals, lower patients' out-of-pocket costs for hospital stays, enhance primary care, and finally revise tariffs for healthcare services to more reasonable rates. Our results speak to the fact that the basic insurance coverage has very little to do with the healthcare costs of SM-exposed people, implying that HSEP's goals are not sufficiently fulfilled for these people. A major obstacle to the effectiveness of the goals might be the healthcare stakeholders' worries.

Our results also show that the potential healthcare needs of SM-exposed people (especially severely injured ones) can lead to regressive payments in the future. On the other side, the history of UHC programs in Iran has shown that attempts to increase access to health insurance have the potential to undermine financial justice by regressively paying for healthcare. People's contributions to new initiatives should grow progressively in line with their income and wealth to provide stable funding and improve equity. In other words, future programs should adjust the healthcare financing to reduce the inequity according to certain neglected vulnerable groups. We believe that the ideal funding mechanism would be a targeted progressive tax based on wealth status. However, this is unrealistic in the near term due to the catastrophic economic level of the general population in recent years [48]. A gradual rise in the progressiveness of finance must be implemented instead.

Our study has a few potential limitations that should be minded when interpreting the results. First, since we did not measure the individual-level socioeconomic determinants of the survivors, it is hard to judge whether the caregivers' personal socioeconomic status is directly associated with HCU. Second, in this study, we focused on the costs paid by insurance companies for caregiving; however, some evidence suggests that indirect expenditures (e.g., for traveling to well-equipped health centers) may also induce a higher burden on survivors and their families. Third, we didn't check whether the SM survivors had formal training in caring. It is becoming more evident that years of education is a helpful index for examining the provincial-level connection with HCU-related factors, yet despite this, it is typically overlooked and not included in policy decisions. More research must be conducted to learn what survivors know about the healthcare services and facilities they need. Moreover, we could not access a group of determinants assumed to aggravate the complications of SM-exposed people and we plan to examine their association in future studies.

## Conclusions

In general, this study is the first to extract the socioeconomic inequalities among SM-exposed survivors on both individual and subnational scales. Poor, less educated and least urbanized areas significantly worse in receiving HCU than prospering regions. Taken together, HCU of SM-exposed survivors is spatially clustered shows significant pro-rich inequity based on most determinants. The findings of the study can role as a guideline for health policymakers in Iran to promote their UHC plans and allocate resources in a more appropriate manner to finally reduce the inequity gaps.

### Supplementary Information

**Additional file 1: Supplementary Table 1. **Healthcare utilization status of the population residing in every province of Iran. 

**Additional file 2: Supplementary Table 2. **Healthcare costs of the population residing in every province of Iran.

**Additional file 3: Supplementary Table 3. **Healthcare utilization status of the population in different months (Nov, 2018 to Jun, 2021).

**Additional file 4: Supplementary Table 4. **Healthcare costs of the population in different months (Nov, 2018 to Jun, 2021).

**Additional file 5: Supplementary Table 5. **Predicted healthcare utilization and related costs per one person in upcoming months (Jul, 2021 to Jun, 2026).

## Data Availability

The datasets generated and/or analysed during the current study are not publicly available due to the regulations of the Veterans and Martyrs Affairs Foundation (VMAF) but are available from the corresponding author on reasonable request.

## Abbreviations

SM: Sulfur Mustard
UHC: Universal Health Coverage
HCU: Healthcare utilization
SDG: Sustainable Development Goals
GGHE-D: Domestic general government health expenditure
USD: United states Dollar
VMAF: Veterans and Martyrs Affairs Foundation
HIES: Households Income and Expenditure Survey
WI: Wealth index
YOS: Years of schooling
LE: Life expectancy
LOS: Length of stay
BOR: Bed occupancy rate
ZIP: Zero-inflated Poisson
