# Supplementary material for: Social disparities and inequalities in healthcare access and expenditures among Iranians exposed to sulfur mustard: a national study using spatio-temporal analysis
Source: BMC Health Serv Res. 2023 Dec 13;23:1406. doi: 10.1186/s12913-023-10352-7 (PMC10720241; doi:10.1186/s12913-023-10352-7)
Supplement: Supplementary file 1 — Additional file 1: Supplementary Table 1. Healthcare utilization status of the population residing in every province of Iran. [file 12913_2023_10352_MOESM1_ESM.docx]

**Supplementary Table 1**: Healthcare utilization status of the population residing in every province of Iran.

| **Province** | **No. of exposed people** | **No. of exposed people benefiting from HCU** | **No. of services** | **HCU rate (per one person)** | | **HCU rate (per one person benefiting from HCU)** | |
| --- | --- | --- | --- | --- | --- | --- | --- |
|  |  |  |  | **Mean (SD)** | **95% CI** | **Mean (SD)** | **95% CI** |
| Alborz | 1,080 | 652 | 20,465 | 19 (35.13) | 16.9-21.1 | 31.39 (40.63) | 28.27-34.51 |
| Ardabil | 789 | 601 | 32,501 | 41.19 (48.24) | 37.83-44.56 | 54.08 (48.56) | 50.2-57.96 |
| Azerbaijan, East | 1,649 | 1,118 | 56,621 | 34.34 (48.8) | 31.98-36.69 | 50.64 (51.83) | 47.61-53.68 |
| Azerbaijan, West | 1,721 | 1,474 | 75,074 | 43.65 (51.48) | 41.21-46.08 | 50.93 (52.17) | 48.27-53.6 |
| Bushehr | 843 | 540 | 26,013 | 30.89 (45.83) | 27.8-33.99 | 48.17 (49.43) | 44-52.34 |
| Chahar Mahaal and Bakhtiari | 1,005 | 786 | 47,405 | 47.17 (51.35) | 43.99-50.34 | 60.31 (50.78) | 56.76-63.86 |
| Fars | 3,919 | 2,509 | 95,736 | 24.45 (37.09) | 23.29-25.61 | 38.16 (40.3) | 36.58-39.73 |
| Gilan | 1,558 | 1,037 | 57,585 | 36.96 (56.54) | 34.15-39.77 | 55.53 (61.41) | 51.79-59.27 |
| Golestan | 1,540 | 1,138 | 60,303 | 39.16 (49.94) | 36.66-41.65 | 52.99 (51.4) | 50-55.98 |
| Hamadan | 1,934 | 1,516 | 79,124 | 40.93 (48.4) | 38.78-43.09 | 52.19 (48.99) | 49.73-54.66 |
| Hormozgan | 212 | 139 | 5,391 | 25.43 (41.52) | 19.84-31.02 | 38.78 (45.97) | 31.14-46.43 |
| Ilam | 745 | 550 | 30,447 | 40.87 (50.06) | 37.27-44.46 | 55.36 (50.92) | 51.1-59.61 |
| Isfahan | 5,787 | 3,891 | 214,045 | 37.01 (55.25) | 35.59-38.44 | 55.01 (59.56) | 53.14-56.88 |
| Kerman | 3,782 | 2,411 | 100,034 | 26.49 (44.83) | 25.06-27.92 | 41.49 (50.26) | 39.48-43.5 |
| Kermanshah | 1,819 | 1,300 | 53,196 | 29.28 (39.38) | 27.47-31.09 | 40.92 (41.12) | 38.68-43.16 |
| Khorasan, North | 687 | 497 | 17,533 | 25.52 (39.91) | 22.54-28.51 | 35.28 (43.11) | 31.49-39.07 |
| Khorasan, Razavi | 3,877 | 2,921 | 109,540 | 28.25 (38.1) | 27.05-29.45 | 37.5 (39.75) | 36.06-38.94 |
| Khorasan, South | 392 | 280 | 7,451 | 19.01 (24.98) | 16.53-21.48 | 26.61 (25.91) | 23.58-29.65 |
| Khuzestan | 3,346 | 1,980 | 90,131 | 26.99 (44.06) | 25.49-28.48 | 45.52 (49.3) | 43.35-47.69 |
| Kohgiluyeh and Boyer-Ahmad | 2,409 | 1,791 | 109,182 | 45.34 (51.64) | 43.28-47.4 | 60.96 (51.31) | 58.59-63.34 |
| Kurdistan | 831 | 680 | 20,795 | 25.08 (32.7) | 22.86-27.31 | 30.58 (33.7) | 28.05-33.11 |
| Lorestan | 1,118 | 768 | 44,508 | 39.81 (49.45) | 36.91-42.71 | 57.95 (50.08) | 54.41-61.49 |
| Markazi | 1,836 | 1,302 | 49,939 | 27.26 (39.76) | 25.44-29.08 | 38.36 (42.41) | 36.05-40.66 |
| Mazandaran | 4,094 | 2,938 | 94,417 | 23.1 (33.26) | 22.08-24.12 | 32.14 (35.34) | 30.86-33.41 |
| Qazvin | 515 | 372 | 17,863 | 34.75 (51) | 30.34-39.16 | 48.02 (54.39) | 42.49-53.55 |
| Qom | 1,552 | 1,127 | 58,401 | 37.63 (50.34) | 35.12-40.13 | 51.82 (52.49) | 48.76-54.88 |
| Semnan | 1,241 | 806 | 35,545 | 28.64 (43.19) | 26.24-31.05 | 44.1 (46.8) | 40.87-47.33 |
| Sistan and Baluchistan | 455 | 243 | 6,914 | 15.2 (26.45) | 12.77-17.63 | 28.45 (30.56) | 24.61-32.29 |
| Tehran | 6,751 | 3,540 | 127,897 | 18.98 (37.41) | 18.09-19.87 | 36.13 (45.22) | 34.64-37.62 |
| Yazd | 964 | 715 | 40,739 | 42.26 (56.4) | 38.7-45.82 | 56.98 (58.74) | 52.67-61.28 |
| Zanjan | 429 | 324 | 19,355 | 45.12 (56.91) | 39.73-50.5 | 59.74 (58.44) | 53.37-66.1 |
| **National (Iran)** | 58,880 | 39,946 | 1,804,150 | 30.67 (45.51) | 30.3-31.04 | 45.16 (48.94) | 44.68-45.64 |
