# Supplementary material for: Social disparities and inequalities in healthcare access and expenditures among Iranians exposed to sulfur mustard: a national study using spatio-temporal analysis
Source: BMC Health Serv Res. 2023 Dec 13;23:1406. doi: 10.1186/s12913-023-10352-7 (PMC10720241; doi:10.1186/s12913-023-10352-7)
Supplement: Supplementary file 2 — Additional file 2: Supplementary Table 2. Healthcare costs of the population residing in every province of Iran. [file 12913_2023_10352_MOESM2_ESM.docx]

**Supplementary Table 2**: Healthcare costs of the population residing in every province of Iran.

| **Province** | **Costs per one health service (USD)** | | | **Costs per one person (USD)** | | | **Costs per one person benefiting from health services (USD)** | | |
| --- | --- | --- | --- | --- | --- | --- | --- | --- | --- |
|  | **Basic insurance** | **Supplementary insurance** | **Total** | **Basic insurance** | **Supplementary insurance** | **Total** | **Basic insurance** | **Supplementary insurance** | **Total** |
| Alborz | 10.82 (9.67-11.96) | 80.06 (71.37-88.76) | 93.54 (84.5-102.57) | 205.51 (161.67-249.36) | 1521.38 (1193.58-1849.19) | 1777.36 (1419.21-2135.5) | 339.47 (268.9-410.05) | 2513.08 (1985.18-3040.98) | 2935.91 (2361.34-3510.48) |
| Ardabil | 13.82 (6.62-21.01) | 20.9 (19.32-22.48) | 36.06 (28.55-43.57) | 569.12 (267.24-871.01) | 860.9 (722.83-998.97) | 1485.51 (1132.6-1838.42) | 747.15 (351.83-1142.48) | 1130.2 (954.36-1306.03) | 1950.19 (1493.1-2407.29) |
| Azerbaijan, East | 8.06 (7.21-8.9) | 24.3 (22.31-26.29) | 34 (31.48-36.51) | 276.59 (231.36-321.83) | 834.27 (724.44-944.1) | 1167.31 (1015.33-1319.29) | 407.96 (342.63-473.3) | 1230.51 (1073.75-1387.27) | 1721.73 (1504.98-1938.48) |
| Azerbaijan, West | 6.67 (6.04-7.3) | 24.79 (22.81-26.77) | 33.13 (30.89-35.36) | 291 (249.93-332.07) | 1081.9 (937.05-1226.75) | 1445.89 (1280.52-1611.27) | 339.57 (292.09-387.04) | 1262.46 (1095.19-1429.73) | 1687.2 (1496.99-1877.41) |
| Bushehr | 10.44 (9.1-11.78) | 23.85 (20.61-27.08) | 37.37 (33.43-41.32) | 322.52 (250.94-394.11) | 736.69 (591.62-881.75) | 1154.64 (950.49-1358.78) | 502.9 (394.18-611.62) | 1148.68 (929.99-1367.38) | 1800.38 (1495.25-2105.5) |
| Chahar Mahaal and Bakhtiari | 12.73 (11.34-14.12) | 17.47 (16.64-18.29) | 31.16 (29.12-33.2) | 600.48 (442.89-758.07) | 823.85 (734.01-913.7) | 1469.82 (1242.25-1697.38) | 767.79 (567.83-967.74) | 1053.4 (943.78-1163.02) | 1879.35 (1594.88-2163.81) |
| Fars | 9.56 (8.8-10.31) | 26.37 (24.53-28.2) | 38.81 (36.54-41.07) | 233.66 (207.12-260.2) | 644.61 (560.41-728.81) | 948.69 (845.99-1051.38) | 364.7 (324.16-405.23) | 1006.09 (876.8-1135.38) | 1480.69 (1324.21-1637.18) |
| Gilan | 9.83 (8.96-10.69) | 27.64 (25.81-29.46) | 39.37 (36.84-41.9) | 363.16 (295-431.32) | 1021.47 (871.48-1171.47) | 1455.15 (1252.69-1657.62) | 545.61 (445.01-646.22) | 1534.67 (1315.85-1753.49) | 2186.24 (1891.9-2480.57) |
| Golestan | 11.67 (10.8-12.54) | 23.94 (21.54-26.35) | 36.25 (33.48-39.02) | 457.06 (393.71-520.4) | 937.55 (804.53-1070.58) | 1419.38 (1244.54-1594.22) | 618.51 (534.76-702.26) | 1268.74 (1092.7-1444.79) | 1920.78 (1691.13-2150.43) |
| Hamadan | 9.37 (8.79-9.95) | 23.45 (21.32-25.58) | 34.19 (31.89-36.5) | 383.41 (339.65-427.16) | 959.99 (819.25-1100.74) | 1399.61 (1231.14-1568.07) | 488.87 (434.26-543.48) | 1224.05 (1046.88-1401.23) | 1784.59 (1573.87-1995.32) |
| Hormozgan | 10.05 (7.1-12.99) | 37.12 (29.3-44.95) | 49.05 (39.42-58.68) | 255.49 (142.78-368.19) | 944 (590.99-1297.01) | 1247.39 (809.15-1685.64) | 389.66 (221.84-557.49) | 1439.77 (919.45-1960.1) | 1902.5 (1259.7-2545.3) |
| Ilam | 8.38 (7.45-9.3) | 26.86 (23.87-29.84) | 36.43 (33.07-39.79) | 342.37 (275.69-409.06) | 1097.57 (911.34-1283.8) | 1488.84 (1258.14-1719.55) | 463.76 (375.62-551.9) | 1486.71 (1242.55-1730.88) | 2016.7 (1716.28-2317.13) |
| Isfahan | 9.42 (8.85-9.99) | 21.97 (21.15-22.79) | 32.54 (31.38-33.7) | 348.69 (316.21-381.18) | 813.26 (740.49-886.03) | 1204.42 (1108.74-1300.11) | 518.24 (470.87-565.62) | 1208.71 (1102.76-1314.66) | 1790.07 (1651.54-1928.61) |
| Kerman | 7.65 (7.16-8.15) | 23.19 (22.13-24.25) | 32.85 (31.32-34.37) | 202.7 (177.59-227.8) | 614.37 (560.86-667.88) | 870.23 (791.69-948.78) | 317.45 (278.87-356.03) | 962.2 (881.64-1042.76) | 1362.92 (1244.32-1481.51) |
| Kermanshah | 8.02 (7.13-8.91) | 24.58 (23.2-25.96) | 34.73 (32.83-36.64) | 234.75 (189.52-279.99) | 719.56 (642.95-796.16) | 1016.9 (908.58-1125.21) | 328.11 (265.61-390.62) | 1005.72 (902.68-1108.75) | 1421.31 (1275.62-1566.99) |
| Khorasan, North | 6.5 (5.52-7.48) | 17.93 (15.92-19.93) | 26.03 (23.31-28.76) | 165.9 (125.11-206.68) | 457.58 (337.31-577.84) | 664.41 (507.73-821.09) | 229.32 (173.93-284.71) | 632.5 (468.81-796.19) | 918.41 (705.98-1130.84) |
| Khorasan, Razavi | 7.9 (7.46-8.34) | 24.04 (22.57-25.51) | 33.84 (32.09-35.59) | 223.18 (203.64-242.71) | 679.24 (616.17-742.32) | 956.06 (877.79-1034.33) | 296.22 (270.85-321.59) | 901.55 (819.41-983.68) | 1268.96 (1167.62-1370.31) |
| Khorasan, South | 7.89 (6.21-9.56) | 20.25 (18.06-22.44) | 29.39 (25.93-32.86) | 149.9 (104.02-195.79) | 384.93 (303.62-466.25) | 558.72 (440.22-677.22) | 209.87 (146.96-272.77) | 538.91 (430.14-647.67) | 782.21 (623.64-940.78) |
| Khuzestan | 8.96 (8.38-9.55) | 32.99 (31.23-34.75) | 43.7 (41.62-45.78) | 241.86 (201.99-281.73) | 890.22 (794.25-986.18) | 1179.26 (1056.31-1302.2) | 407.99 (341.71-474.26) | 1501.68 (1345.39-1657.97) | 1989.25 (1789.51-2188.99) |
| Kohgiluyeh and Boyer-Ahmad | 9.13 (8.54-9.73) | 17.62 (16.71-18.53) | 28.19 (26.83-29.54) | 414.16 (372.13-456.2) | 798.84 (729.93-867.75) | 1278.12 (1170.55-1385.69) | 556.84 (501.85-611.83) | 1074.04 (984.87-1163.21) | 1718.44 (1579.52-1857.35) |
| Kurdistan | 8 (7-9) | 29.56 (26.61-32.51) | 40.24 (36.77-43.71) | 200.63 (157.07-244.18) | 741.56 (582.76-900.35) | 1009.44 (818.73-1200.14) | 244.59 (192.06-297.12) | 904.05 (712.59-1095.51) | 1230.62 (1001.44-1459.81) |
| Lorestan | 9.28 (8.27-10.29) | 33.53 (28.1-38.96) | 44 (38.15-49.85) | 369.56 (305.12-434) | 1334.77 (1045.42-1624.12) | 1751.66 (1416.14-2087.17) | 537.98 (446.6-629.36) | 1943.06 (1528.84-2357.28) | 2549.94 (2071.97-3027.9) |
| Markazi | 6.95 (6.14-7.77) | 30.69 (28.54-32.84) | 39.07 (36.42-41.71) | 189.58 (157.97-221.18) | 836.66 (727.71-945.6) | 1064.9 (930.69-1199.1) | 266.75 (222.96-310.54) | 1177.23 (1027.84-1326.62) | 1498.38 (1314.67-1682.09) |
| Mazandaran | 13.91 (13.09-14.73) | 37.37 (35.15-39.6) | 54.5 (50.89-58.11) | 321.24 (287.88-354.59) | 863.21 (781.95-944.47) | 1258.67 (1135.69-1381.65) | 446.98 (401.37-492.59) | 1201.09 (1090.39-1311.79) | 1751.34 (1583.54-1919.14) |
| Qazvin | 9.25 (7.62-10.88) | 45.92 (28.44-63.4) | 57.13 (39.49-74.78) | 321.56 (230.06-413.06) | 1595.74 (846.6-2344.87) | 1985.6 (1191.48-2779.72) | 444.31 (320.08-568.53) | 2204.86 (1176.12-3233.6) | 2743.55 (1655.74-3831.35) |
| Qom | 7.43 (6.8-8.05) | 25.78 (24.25-27.31) | 34.4 (32.55-36.25) | 279.4 (236.52-322.29) | 970.21 (846.31-1094.11) | 1294.49 (1137.87-1451.11) | 384.77 (326.88-442.66) | 1336.09 (1170.4-1501.77) | 1782.65 (1573.93-1991.36) |
| Semnan | 9.75 (8.5-10.99) | 22.24 (19.92-24.56) | 33.22 (30.35-36.09) | 279.21 (230.24-328.18) | 636.89 (541.53-732.26) | 951.48 (822.3-1080.65) | 429.9 (356.57-503.23) | 980.62 (839.34-1121.9) | 1464.99 (1275.3-1654.68) |
| Sistan and Baluchistan | 9.38 (7.18-11.57) | 34.79 (21.01-48.57) | 45.8 (31.22-60.38) | 142.5 (97.46-187.54) | 528.65 (253.82-803.49) | 696.01 (385.65-1006.38) | 266.82 (185.59-348.06) | 989.86 (481.84-1497.89) | 1303.24 (732.44-1874.03) |
| Tehran | 9.87 (9.36-10.38) | 64.61 (61.74-67.47) | 77.51 (74.39-80.64) | 187.31 (165.46-209.17) | 1226.14 (1128.76-1323.51) | 1471.11 (1357.17-1585.05) | 356.58 (315.77-397.39) | 2334.16 (2156.51-2511.81) | 2800.51 (2593.12-3007.9) |
| Yazd | 8.17 (7.54-8.81) | 21.02 (19.76-22.28) | 30.66 (28.79-32.52) | 345.45 (296-394.89) | 888.4 (731.48-1045.31) | 1295.52 (1096.77-1494.28) | 465.75 (401.38-530.12) | 1197.78 (990.95-1404.61) | 1746.69 (1486.7-2006.69) |
| Zanjan | 11.28 (9.77-12.79) | 26.92 (23.39-30.44) | 39.8 (35.6-44) | 508.79 (334.71-682.86) | 1214.33 (903.3-1525.35) | 1795.71 (1380.2-2211.22) | 673.67 (445.98-901.37) | 1607.86 (1205.11-2010.6) | 2377.65 (1842.43-2912.87) |
| **National (Iran)** | 9.34 (9.14-9.54) | 28.78 (28.33-29.24) | 39.93 (39.38-40.49) | 286.47 (277.22-295.73) | 882.71 (858.9-906.53) | 1224.67 (1194.62-1254.72) | 421.88 (408.45-435.31) | 1299.94 (1265.62-1334.26) | 1803.53 (1760.42-1846.63) |
