# Supplementary material for: Social disparities and inequalities in healthcare access and expenditures among Iranians exposed to sulfur mustard: a national study using spatio-temporal analysis
Source: BMC Health Serv Res. 2023 Dec 13;23:1406. doi: 10.1186/s12913-023-10352-7 (PMC10720241; doi:10.1186/s12913-023-10352-7)
Supplement: Supplementary file 3 — Additional file 3: Supplementary Table 3. Healthcare utilization status of the population in different months (Nov, 2018 to Jun, 2021). [file 12913_2023_10352_MOESM3_ESM.docx]

**Supplementary Table 3**: Healthcare utilization status of the population in different months (Nov, 2018 to Jun, 2021).

| **Month, year** | **No. of exposed people benefiting from HCU** | **No. of services** | **HCU rate (per one person)** | | **HCU rate (per one person benefiting from HCU)** | |
| --- | --- | --- | --- | --- | --- | --- |
|  |  |  | **Mean (SD)** | **95% CI** | **Mean (SD)** | **95% CI** |
| Nov, 2018 | 13,840 | 49,671 | 0.84 (2.14) | 0.83-0.86 | 3.59 (3.09) | 3.54-3.64 |
| Dec, 2018 | 13,776 | 50,385 | 0.86 (2.23) | 0.84-0.87 | 3.66 (3.31) | 3.6-3.71 |
| Jan, 2019 | 14,752 | 56,727 | 0.96 (2.4) | 0.94-0.98 | 3.85 (3.44) | 3.79-3.9 |
| Feb, 2019 | 15,986 | 58,590 | 1 (2.33) | 0.98-1.01 | 3.67 (3.2) | 3.62-3.71 |
| Mar, 2019 | 16,048 | 57,857 | 0.98 (2.3) | 0.96-1 | 3.61 (3.15) | 3.56-3.65 |
| Apr, 2019 | 13,238 | 41,549 | 0.71 (1.84) | 0.69-0.72 | 3.14 (2.73) | 3.09-3.19 |
| May, 2019 | 15,813 | 57,968 | 0.99 (2.34) | 0.97-1.01 | 3.67 (3.26) | 3.62-3.72 |
| Jun, 2019 | 14,939 | 50,746 | 0.87 (2.11) | 0.85-0.88 | 3.4 (3) | 3.35-3.44 |
| Jul, 2019 | 15,973 | 59,351 | 1.01 (2.46) | 0.99-1.03 | 3.72 (3.49) | 3.66-3.77 |
| Aug, 2019 | 14,878 | 52,563 | 0.9 (2.24) | 0.88-0.92 | 3.53 (3.25) | 3.48-3.59 |
| Sep, 2019 | 15,157 | 53,506 | 0.91 (2.24) | 0.9-0.93 | 3.53 (3.19) | 3.48-3.58 |
| Oct, 2019 | 15,296 | 53,539 | 0.91 (2.2) | 0.9-0.93 | 3.5 (3.1) | 3.45-3.55 |
| Nov, 2019 | 14,435 | 47,867 | 0.82 (2.08) | 0.8-0.83 | 3.32 (3.05) | 3.27-3.37 |
| Dec, 2019 | 19,440 | 70,710 | 1.21 (2.53) | 1.19-1.23 | 3.64 (3.25) | 3.59-3.68 |
| Jan, 1398 | 18,996 | 66,417 | 1.13 (2.4) | 1.11-1.15 | 3.5 (3.1) | 3.45-3.54 |
| Feb, 2020 | 18,697 | 64,317 | 1.1 (2.35) | 1.08-1.12 | 3.44 (3.06) | 3.4-3.48 |
| Mar, 2020 | 16,409 | 51,515 | 0.88 (2.03) | 0.86-0.9 | 3.14 (2.78) | 3.1-3.18 |
| Apr, 2020 | 12,797 | 36,661 | 0.63 (1.71) | 0.61-0.64 | 2.86 (2.64) | 2.82-2.91 |
| May, 2020 | 15,842 | 54,860 | 0.94 (2.24) | 0.92-0.96 | 3.46 (3.13) | 3.41-3.51 |
| Jun, 2020 | 16,002 | 53,596 | 0.92 (2.18) | 0.9-0.93 | 3.35 (3.05) | 3.3-3.4 |
| Jul, 2020 | 17,437 | 62,137 | 1.06 (2.39) | 1.04-1.08 | 3.56 (3.23) | 3.52-3.61 |
| Aug, 2020 | 16,916 | 58,859 | 1.01 (2.33) | 0.99-1.03 | 3.48 (3.2) | 3.43-3.53 |
| Sep, 2020 | 17,092 | 61,538 | 1.05 (2.43) | 1.03-1.07 | 3.6 (3.35) | 3.55-3.65 |
| Oct, 2020 | 17,619 | 62,224 | 1.06 (2.42) | 1.04-1.08 | 3.53 (3.29) | 3.48-3.58 |
| Nov, 2020 | 17,671 | 61,707 | 1.06 (2.39) | 1.04-1.07 | 3.49 (3.23) | 3.44-3.54 |
| Dec, 2020 | 17,055 | 60,345 | 1.03 (2.38) | 1.01-1.05 | 3.54 (3.26) | 3.49-3.59 |
| Jan, 2020 | 17,562 | 62,441 | 1.07 (2.37) | 1.05-1.09 | 3.56 (3.15) | 3.51-3.6 |
| Feb, 2021 | 18,467 | 66,957 | 1.15 (2.48) | 1.13-1.17 | 3.63 (3.27) | 3.58-3.67 |
| Mar, 2021 | 17,793 | 60,792 | 1.04 (2.27) | 1.02-1.06 | 3.42 (2.99) | 3.37-3.46 |
| Apr, 2021 | 15,448 | 50,068 | 0.86 (2.08) | 0.84-0.88 | 3.24 (2.95) | 3.19-3.29 |
| May, 2021 | 16,110 | 54,411 | 0.93 (2.24) | 0.91-0.95 | 3.38 (3.17) | 3.33-3.43 |
| Jun, 2021 | 16,380 | 54,276 | 0.93 (2.2) | 0.91-0.95 | 3.31 (3.07) | 3.27-3.36 |
| **Monthly** | 16,183 | 56,380 | 0.96 (2.26) | 0.94-0.98 | 3.48 (3.14) | 3.43-3.53 |
| **Total** | 39,946 | 1,804,150 | 30.67 (45.51) | 30.3-31.04 | 45.16 (48.94) | 44.68-45.64 |
