# Supplementary material for: Social disparities and inequalities in healthcare access and expenditures among Iranians exposed to sulfur mustard: a national study using spatio-temporal analysis
Source: BMC Health Serv Res. 2023 Dec 13;23:1406. doi: 10.1186/s12913-023-10352-7 (PMC10720241; doi:10.1186/s12913-023-10352-7)
Supplement: Supplementary file 4 — Additional file 4: Supplementary Table 4. Healthcare costs of the population in different months (Nov, 2018 to Jun, 2021). [file 12913_2023_10352_MOESM4_ESM.docx]

**Supplementary Table 4**: Healthcare costs of the population in different months (Nov, 2018 to Jun, 2021).

| **Month, year** | **Costs per one health service (USD)** | | | **Costs per one person (USD)** | | | **Costs per one person benefiting from health services (USD)** | | |
| --- | --- | --- | --- | --- | --- | --- | --- | --- | --- |
|  | **Basic insurance** | **Supplementary insurance** | **Total** | **Basic insurance** | **Supplementary insurance** | **Total** | **Basic insurance** | **Supplementary insurance** | **Total** |
| Nov, 2018 | 6.99 (6.26-7.71) | 22.62 (19.83-25.41) | 31.64 (26.12-37.16) | 5.89 (5.25-6.54) | 19.08 (16.63-21.53) | 26.69 (21.96-31.42) | 25.08 (22.35-27.8) | 81.17 (70.81-91.53) | 113.55 (93.49-133.62) |
| Dec, 2018 | 6.97 (6.47-7.47) | 22.06 (20.47-23.65) | 29.53 (27.67-31.39) | 5.97 (5.5-6.44) | 18.88 (17.35-20.41) | 25.27 (23.47-27.07) | 25.51 (23.53-27.48) | 80.68 (74.25-87.11) | 108 (100.47-115.53) |
| Jan, 2019 | 7.83 (7.18-8.48) | 24.16 (22.53-25.8) | 32.32 (30.36-34.29) | 7.54 (6.86-8.22) | 23.28 (21.45-25.11) | 31.14 (28.92-33.36) | 30.1 (27.43-32.78) | 92.92 (85.71-100.12) | 124.29 (115.62-132.97) |
| Feb, 2019 | 7.55 (6.97-8.13) | 23.76 (21.44-26.07) | 31.9 (29.37-34.43) | 7.51 (6.91-8.12) | 23.64 (21.2-26.09) | 31.74 (29.03-34.46) | 27.68 (25.48-29.88) | 87.08 (78.15-96.02) | 116.92 (107.04-126.79) |
| Mar, 2019 | 7.07 (6.5-7.63) | 21.9 (20.23-23.58) | 29.77 (27.78-31.77) | 6.94 (6.36-7.53) | 21.52 (19.72-23.33) | 29.26 (26.94-31.57) | 25.47 (23.35-27.6) | 78.97 (72.43-85.52) | 107.34 (98.96-115.73) |
| Apr, 2019 | 7.73 (6.99-8.47) | 20.45 (18.38-22.51) | 29.09 (26.61-31.57) | 5.46 (4.91-6) | 14.43 (12.81-16.04) | 20.53 (18.58-22.47) | 24.28 (21.88-26.67) | 64.17 (57.05-71.3) | 91.31 (82.77-99.84) |
| May, 2019 | 8.04 (7.45-8.63) | 22.88 (21.29-24.46) | 31.42 (29.59-33.26) | 7.91 (7.29-8.54) | 22.52 (20.68-24.36) | 30.94 (28.82-33.06) | 29.46 (27.17-31.75) | 83.86 (77.1-90.62) | 115.19 (107.45-122.93) |
| Jun, 2019 | 9.07 (8.22-9.93) | 26.46 (23.21-29.71) | 36.44 (32.86-40.01) | 7.82 (7.03-8.61) | 22.8 (19.82-25.79) | 31.4 (28.04-34.76) | 30.82 (27.75-33.89) | 89.88 (78.17-101.59) | 123.77 (110.64-136.89) |
| Jul, 2019 | 8.52 (7.72-9.32) | 24.33 (22.63-26.04) | 33.77 (31.63-35.9) | 8.59 (7.74-9.44) | 24.53 (22.62-26.43) | 34.04 (31.64-36.43) | 31.65 (28.54-34.75) | 90.41 (83.5-97.32) | 125.46 (116.78-134.14) |
| Aug, 2019 | 8.36 (7.57-9.14) | 25.46 (23.33-27.58) | 35 (32.54-37.45) | 7.46 (6.72-8.2) | 22.73 (20.64-24.81) | 31.24 (28.81-33.67) | 29.53 (26.64-32.42) | 89.94 (81.79-98.08) | 123.64 (114.18-133.11) |
| Sep, 2019 | 8.93 (7.99-9.88) | 23.21 (21.65-24.76) | 33.46 (31.32-35.59) | 8.12 (7.22-9.01) | 21.09 (19.48-22.7) | 30.4 (28.2-32.61) | 31.53 (28.09-34.98) | 81.92 (75.77-88.06) | 118.1 (109.7-126.51) |
| Oct, 2019 | 11.54 (6.98-16.11) | 25.01 (22.53-27.48) | 37.89 (32.61-43.17) | 10.5 (6.34-14.65) | 22.74 (20.37-25.11) | 34.45 (29.55-39.35) | 40.41 (24.41-56.4) | 87.52 (78.48-96.57) | 132.62 (113.85-151.4) |
| Nov, 2019 | 8.44 (7.77-9.11) | 23.4 (21.83-24.97) | 33.1 (31.1-35.09) | 6.86 (6.28-7.45) | 19.02 (17.49-20.56) | 26.91 (24.97-28.84) | 27.99 (25.64-30.35) | 77.6 (71.44-83.75) | 109.75 (102-117.5) |
| Dec, 2019 | 7.94 (7.36-8.52) | 26.77 (24.88-28.66) | 36.56 (34.25-38.86) | 9.53 (8.81-10.26) | 32.15 (29.63-34.67) | 43.9 (40.78-47.02) | 28.88 (26.7-31.05) | 97.38 (89.82-104.94) | 132.96 (123.64-142.28) |
| Jan, 1398 | 9.48 (8.64-10.31) | 27.72 (25.75-29.7) | 38.93 (36.49-41.36) | 10.69 (9.68-11.7) | 31.27 (28.85-33.69) | 43.91 (40.88-46.94) | 33.13 (30.03-36.23) | 96.94 (89.52-104.35) | 136.1 (126.83-145.36) |
| Feb, 2020 | 8.78 (8.12-9.44) | 26.59 (24.92-28.25) | 37.02 (34.99-39.05) | 9.59 (8.8-10.37) | 29.04 (27-31.09) | 40.44 (37.88-42.99) | 30.19 (27.75-32.63) | 91.46 (85.1-97.82) | 127.35 (119.45-135.25) |
| Mar, 2020 | 8.27 (7.49-9.04) | 20.89 (19.17-22.62) | 31.14 (28.78-33.5) | 7.23 (6.5-7.96) | 18.28 (16.49-20.07) | 27.24 (24.8-29.69) | 25.95 (23.34-28.56) | 65.59 (59.23-71.96) | 97.76 (89.07-106.45) |
| Apr, 2020 | 8.87 (7.91-9.83) | 22.75 (18.55-26.96) | 32.55 (28.04-37.06) | 5.52 (4.9-6.14) | 14.17 (11.47-16.86) | 20.27 (17.36-23.17) | 25.4 (22.56-28.24) | 65.19 (52.84-77.54) | 93.26 (79.97-106.54) |
| May, 2020 | 8.96 (8.17-9.75) | 24.45 (22.5-26.4) | 34.93 (32.51-37.36) | 8.35 (7.55-9.15) | 22.78 (20.78-24.78) | 32.55 (30.02-35.08) | 31.04 (28.09-33.99) | 84.67 (77.32-92.03) | 120.98 (111.71-130.25) |
| Jun, 2020 | 8.73 (8.02-9.44) | 27.69 (25.17-30.21) | 38.28 (35.47-41.09) | 7.95 (7.26-8.64) | 25.21 (22.76-27.65) | 34.84 (32.07-37.62) | 29.24 (26.73-31.75) | 92.75 (83.83-101.67) | 128.21 (118.14-138.28) |
| Jul, 2020 | 9.41 (8.68-10.14) | 31.78 (28.4-35.15) | 43.3 (39.61-47) | 9.93 (9.09-10.78) | 33.54 (29.76-37.31) | 45.7 (41.5-49.9) | 33.54 (30.72-36.36) | 113.25 (100.58-125.91) | 154.31 (140.25-168.37) |
| Aug, 2020 | 9.16 (8.36-9.96) | 35.54 (29.93-41.15) | 47.17 (41.34-52.99) | 9.16 (8.32-9.99) | 35.53 (29.74-41.32) | 47.15 (41.01-53.29) | 31.87 (28.98-34.76) | 123.66 (103.56-143.76) | 164.11 (142.85-185.37) |
| Sep, 2020 | 9.99 (8.85-11.14) | 34.47 (31.69-37.25) | 47.03 (43.62-50.43) | 10.44 (9.21-11.68) | 36.03 (32.38-39.67) | 49.15 (44.8-53.5) | 35.97 (31.75-40.2) | 124.1 (111.63-136.57) | 169.32 (154.5-184.15) |
| Oct, 2020 | 9.71 (8.83-10.59) | 32.8 (30.61-34.99) | 45.04 (42.28-47.79) | 10.26 (9.28-11.25) | 34.66 (31.54-37.78) | 47.59 (43.83-51.35) | 34.29 (31.03-37.55) | 115.83 (105.51-126.15) | 159.05 (146.64-171.46) |
| Nov, 2020 | 9.87 (9.02-10.72) | 31.56 (29.44-33.67) | 44.25 (41.57-46.93) | 10.34 (9.4-11.29) | 33.07 (30.56-35.58) | 46.37 (43.16-49.58) | 34.46 (31.34-37.59) | 110.19 (101.94-118.44) | 154.51 (144-165.02) |
| Dec, 2020 | 10.93 (10-11.87) | 31.27 (29.45-33.09) | 44.63 (42.24-47.03) | 11.2 (10.19-12.22) | 32.05 (29.91-34.2) | 45.74 (42.92-48.57) | 38.68 (35.22-42.14) | 110.66 (103.38-117.93) | 157.93 (148.37-167.48) |
| Jan, 2020 | 10.35 (9.5-11.19) | 35.39 (32.67-38.11) | 48.73 (45.63-51.83) | 10.97 (10.01-11.94) | 37.53 (34.21-40.85) | 51.68 (47.67-55.7) | 36.79 (33.59-39.99) | 125.82 (114.81-136.83) | 173.27 (159.98-186.56) |
| Feb, 2021 | 10.24 (9.35-11.13) | 35.76 (33.64-37.87) | 48.67 (46.1-51.24) | 11.64 (10.59-12.69) | 40.66 (37.86-43.46) | 55.34 (51.92-58.77) | 37.13 (33.81-40.45) | 129.64 (120.84-138.45) | 176.46 (165.74-187.18) |
| Mar, 2021 | 10.88 (9.98-11.79) | 39.64 (35.47-43.81) | 53.71 (49.24-58.17) | 11.24 (10.26-12.21) | 40.93 (36.37-45.48) | 55.45 (50.51-60.39) | 37.18 (33.98-40.38) | 135.43 (120.46-150.41) | 183.5 (167.32-199.68) |
| Apr, 2021 | 12.36 (11.21-13.51) | 36.79 (34.24-39.34) | 51.86 (48.55-55.16) | 10.51 (9.5-11.53) | 31.28 (28.88-33.69) | 44.09 (40.97-47.22) | 40.07 (36.24-43.9) | 119.24 (110.22-128.25) | 168.07 (156.39-179.75) |
| May, 2021 | 13.5 (12.28-14.73) | 41.82 (38.97-44.68) | 58.98 (55.27-62.69) | 12.48 (11.3-13.66) | 38.65 (35.55-41.74) | 54.5 (50.49-58.52) | 45.61 (41.33-49.88) | 141.25 (130.08-152.42) | 199.21 (184.76-213.65) |
| Jun, 2021 | 13.67 (12.51-14.82) | 42.13 (39.44-44.83) | 58.17 (54.83-61.52) | 12.6 (11.47-13.73) | 38.84 (35.99-41.69) | 53.62 (50.08-57.17) | 45.28 (41.26-49.31) | 139.6 (129.52-149.69) | 192.76 (180.26-205.26) |
| **Monthly** | 9.32 (8.39-10.25) | 28.48 (26.07-30.9) | 39.57 (36.57-42.57) | 8.94 (8.02-9.87) | 27.56 (25-30.12) | 38.24 (35.05-41.42) | 32.32 (28.96-35.68) | 99.02 (89.84-108.21) | 137.47 (126.03-148.92) |
| **Total** | 9.34 (9.14-9.54) | 28.78 (28.33-29.24) | 39.93 (39.38-40.49) | 286.47 (277.22-295.73) | 882.71 (858.9-906.53) | 1224.67 (1194.62-1254.72) | 421.88 (408.45-435.31) | 1299.94 (1265.62-1334.26) | 1803.53 (1760.42-1846.63) |
