# Supplementary material for: Social disparities and inequalities in healthcare access and expenditures among Iranians exposed to sulfur mustard: a national study using spatio-temporal analysis
Source: BMC Health Serv Res. 2023 Dec 13;23:1406. doi: 10.1186/s12913-023-10352-7 (PMC10720241; doi:10.1186/s12913-023-10352-7)
Supplement: Supplementary file 5 — Additional file 5: Supplementary Table 5. Predicted healthcare utilization and related costs per one person in upcoming months (Jul, 2021 to Jun, 2026). [file 12913_2023_10352_MOESM5_ESM.docx]

**Supplementary Table 5**: Predicted healthcare utilization and related costs per one person in upcoming months (Jul, 2021 to Jun, 2026).

| **Month, year** | **HCU rate (95% CI)** | **Costs in USD (95% CI)** | | | **Month, year** | **HCU rate (95% CI)** | **Costs in USD (95% CI)** | | |
| --- | --- | --- | --- | --- | --- | --- | --- | --- | --- |
|  |  | **Basic insurance** | **Supplementary insurance** | **Total** |  |  | **Basic insurance** | **Supplementary insurance** | **Total** |
| Jul 2021 | 1.15 (1.04-1.27) | 14.44 (11.82-17.65) | 44.47 (36.73-53.85) | 63.54 (53.02-76.16) | Jan 2023 | 1.32 (1.04-1.6) | 33.12 (26.11-42.02) | 106.84 (85.16-134.06) | 157.02 (126.7-194.62) |
| Aug 2021 | 1.07 (0.95-1.19) | 13.27 (10.86-16.22) | 42.62 (35.2-51.61) | 61.34 (51.18-73.52) | Feb 2024 | 1.33 (1.05-1.62) | 32.84 (25.9-41.66) | 109.14 (87-136.93) | 159.66 (128.84-197.87) |
| Sep 2021 | 1.1 (0.97-1.23) | 14.72 (12.04-17.99) | 43.67 (36.06-52.88) | 64.04 (53.43-76.76) | Mar 2024 | 1.22 (0.93-1.51) | 28.84 (22.76-36.55) | 86.42 (68.91-108.38) | 130.56 (105.4-161.76) |
| Oct 2021 | 1.11 (0.97-1.24) | 14.38 (11.77-17.58) | 39.79 (32.86-48.19) | 59.97 (50.04-71.89) | Apr 2024 | 0.98 (0.7-1.28) | 24.31 (19.14-30.86) | 63.69 (50.76-79.93) | 98.97 (79.84-122.69) |
| Nov 2021 | 1.06 (0.92-1.19) | 13.43 (11.03-16.35) | 41.03 (34.03-49.48) | 59.15 (49.54-70.61) | May 2024 | 1.19 (0.89-1.49) | 33.65 (26.5-42.71) | 93.68 (74.66-117.56) | 143.19 (115.52-177.5) |
| Dec 2021 | 1.18 (1.04-1.32) | 15.36 (12.62-18.7) | 49.05 (40.67-59.15) | 70.18 (58.78-83.79) | Jun 2024 | 1.14 (0.84-1.45) | 32.9 (25.92-41.77) | 105.35 (83.96-132.19) | 156.61 (126.35-194.13) |
| Jan 2021 | 1.22 (1.07-1.37) | 17.58 (14.44-21.41) | 55.23 (45.79-66.62) | 79.01 (66.17-94.35) | Jul 2024 | 1.3 (0.99-1.61) | 37.33 (28.8-48.38) | 119.66 (93.44-153.25) | 178.02 (140.88-224.97) |
| Feb 2022 | 1.23 (1.08-1.38) | 17.44 (14.32-21.23) | 56.42 (46.78-68.04) | 80.34 (67.3-95.92) | Aug 2024 | 1.21 (0.9-1.53) | 34.31 (26.48-44.46) | 114.68 (89.55-146.87) | 171.83 (135.98-217.15) |
| Mar 2022 | 1.12 (0.97-1.28) | 15.31 (12.59-18.63) | 44.67 (37.06-53.85) | 65.7 (55.06-78.41) | Sep 2024 | 1.25 (0.93-1.57) | 38.05 (29.37-49.31) | 117.5 (91.76-150.48) | 179.4 (141.97-226.71) |
| Apr 2022 | 0.88 (0.72-1.04) | 12.9 (10.59-15.73) | 32.92 (27.29-39.72) | 49.8 (41.7-59.48) | Oct 2024 | 1.25 (0.93-1.58) | 37.18 (28.7-48.16) | 107.06 (83.61-137.1) | 168.02 (132.98-212.3) |
| May 2022 | 1.09 (0.93-1.26) | 17.86 (14.66-21.76) | 48.43 (40.15-58.42) | 72.05 (60.34-86.05) | Nov 2024 | 1.2 (0.88-1.54) | 34.72 (26.67-45.22) | 110.4 (85.79-142.08) | 165.7 (130.53-210.35) |
| Jun 2022 | 1.05 (0.88-1.22) | 17.47 (14.34-21.28) | 54.46 (45.15-65.68) | 78.81 (66-94.1) | Dec 2024 | 1.33 (0.99-1.67) | 39.7 (30.49-51.71) | 131.97 (102.55-169.85) | 196.6 (154.86-249.6) |
| Jul 2022 | 1.2 (1.02-1.38) | 19.82 (15.98-24.57) | 61.85 (50.38-75.94) | 89.58 (73.78-108.77) | Jan 2024 | 1.37 (1.02-1.71) | 45.45 (34.89-59.22) | 148.61 (115.46-191.29) | 221.35 (174.34-281.07) |
| Aug 2022 | 1.12 (0.93-1.3) | 18.22 (14.69-22.59) | 59.28 (48.29-72.78) | 86.47 (71.21-104.99) | Feb 2025 | 1.38 (1.03-1.73) | 45.07 (34.61-58.72) | 151.8 (117.95-195.39) | 225.07 (177.29-285.77) |
| Sep 2022 | 1.15 (0.96-1.34) | 20.2 (16.29-25.05) | 60.74 (49.47-74.57) | 90.28 (74.35-109.62) | Mar 2025 | 1.27 (0.92-1.63) | 39.58 (30.41-51.52) | 120.2 (93.42-154.66) | 184.06 (145.03-233.62) |
| Oct 2022 | 1.15 (0.96-1.35) | 19.74 (15.92-24.47) | 55.34 (45.08-67.95) | 84.55 (69.63-102.66) | Apr 2025 | 1.03 (0.7-1.39) | 33.36 (25.59-43.5) | 88.59 (68.82-114.05) | 139.52 (109.87-177.19) |
| Nov 2022 | 1.11 (0.91-1.31) | 18.44 (14.87-22.85) | 57.07 (46.51-70.02) | 83.38 (68.71-101.19) | May 2025 | 1.24 (0.88-1.61) | 46.18 (35.42-60.2) | 130.3 (101.22-167.75) | 201.86 (158.96-256.35) |
| Dec 2022 | 1.23 (1.03-1.44) | 21.08 (17.01-26.13) | 68.22 (55.6-83.71) | 98.93 (81.51-120.07) | Jun 2025 | 1.19 (0.84-1.57) | 45.15 (34.64-58.87) | 146.53 (113.83-188.63) | 220.78 (173.86-280.37) |
| Jan 2022 | 1.27 (1.06-1.48) | 24.13 (19.46-29.92) | 76.82 (62.6-94.28) | 111.38 (91.77-135.21) | Jul 2025 | 1.35 (0.98-1.73) | 51.23 (38.46-68.23) | 166.43 (126.6-218.81) | 250.96 (193.74-325.09) |
| Feb 2023 | 1.28 (1.07-1.5) | 23.93 (19.31-29.67) | 78.47 (63.95-96.3) | 113.26 (93.32-137.46) | Aug 2025 | 1.26 (0.89-1.65) | 47.09 (35.36-62.71) | 159.5 (121.33-209.7) | 242.23 (187.01-313.79) |
| Mar 2023 | 1.17 (0.95-1.39) | 21.01 (16.97-26.03) | 62.13 (50.65-76.22) | 92.62 (76.35-112.37) | Sep 2025 | 1.29 (0.92-1.69) | 52.22 (39.22-69.54) | 163.43 (124.33-214.85) | 252.91 (195.25-327.61) |
| Apr 2023 | 0.93 (0.71-1.16) | 17.71 (14.27-21.98) | 45.79 (37.31-56.21) | 70.21 (57.83-85.24) | Oct 2025 | 1.3 (0.92-1.7) | 51.03 (38.34-67.93) | 148.91 (113.29-195.74) | 236.85 (182.88-306.77) |
| May 2023 | 1.14 (0.91-1.38) | 24.52 (19.76-30.41) | 67.36 (54.88-82.67) | 101.57 (83.67-123.31) | Nov 2025 | 1.25 (0.87-1.66) | 47.66 (35.54-63.9) | 153.56 (116.02-203.25) | 233.58 (179.19-304.52) |
| Jun 2023 | 1.1 (0.86-1.33) | 23.97 (19.33-29.74) | 75.74 (61.72-92.96) | 111.09 (91.52-134.86) | Dec 2025 | 1.38 (0.98-1.79) | 54.49 (40.64-73.08) | 183.55 (138.68-242.98) | 277.14 (212.59-361.34) |
| Jul 2023 | 1.25 (1.01-1.5) | 27.2 (21.5-34.41) | 86.03 (68.75-107.66) | 126.28 (102.14-156.13) | Jan 2025 | 1.42 (1.01-1.83) | 62.37 (46.5-83.68) | 206.69 (156.14-273.65) | 312.04 (239.32-406.9) |
| Aug 2023 | 1.17 (0.92-1.42) | 25 (19.77-31.62) | 82.45 (65.89-103.18) | 121.89 (98.6-150.71) | Feb 2026 | 1.43 (1.02-1.85) | 61.86 (46.13-82.98) | 211.14 (159.5-279.52) | 317.29 (243.36-413.71) |
| Sep 2023 | 1.2 (0.94-1.45) | 27.73 (21.92-35.07) | 84.48 (67.51-105.72) | 127.26 (102.94-157.35) | Mar 2026 | 1.32 (0.91-1.75) | 54.32 (40.53-72.82) | 167.18 (126.33-221.26) | 259.47 (199.08-338.21) |
| Oct 2023 | 1.2 (0.94-1.47) | 27.09 (21.42-34.26) | 76.97 (61.52-96.32) | 119.18 (96.41-147.35) | Apr 2026 | 1.08 (0.71-1.51) | 45.78 (34.1-61.47) | 123.22 (93.07-163.15) | 196.69 (150.83-256.51) |
| Nov 2023 | 1.16 (0.89-1.42) | 25.3 (19.95-32.09) | 79.37 (63.28-99.57) | 117.54 (94.86-145.65) | May 2026 | 1.29 (0.88-1.73) | 63.37 (47.2-85.08) | 181.24 (136.88-239.97) | 284.56 (218.2-371.11) |
| Dec 2023 | 1.28 (1.01-1.55) | 28.93 (22.81-36.69) | 94.88 (75.64-119.04) | 139.46 (112.54-172.83) | Jun 2026 | 1.24 (0.84-1.69) | 61.97 (46.16-83.2) | 203.81 (153.93-269.86) | 311.23 (238.66-405.89) |
